# Dynamic interplay of cNHEJ and MMEJ pathways of DNA double-strand break repair during embryonic development in zebrafish

**DOI:** 10.1038/s41598-025-88564-6

**Published:** 2025-02-10

**Authors:** Mathieu Carrara, Anne-Laure Gaillard, Alice Brion, Evelyne Duvernois-Berthet, Carine Giovannangeli, Jean-Paul Concordet, Guillaume Pézeron

**Affiliations:** 1 Physiologie Moléculaire et Adaptation (PhyMA, UMR7221), Muséum national d’Histoire naturelle, CNRS, Paris, France; 2 Structure and Instability of Genomes Laboratory (StrING UMR7196 - U1154), Muséum national d’Histoire naturelle, CNRS, INSERM, Paris, France

**Keywords:** DNA repair, MMEJ, cNHEJ, Zebrafish, DNA damage and repair, Embryogenesis

## Abstract

Double strand breaks (DSBs) are the most deleterious DNA lesions as they frequently result in mutations when repaired by canonical non homologous end-joining (cNHEJ) and microhomology-mediated end-joining (MMEJ). Here, we investigated the relative importance of cNHEJ and MMEJ pathways during zebrafish embryonic development. We have analyzed the expression of cNHEJ and MMEJ related genes and found that it was dynamic during development and often become increased in specific tissues. We showed that inactivation of nuclear DNA ligase 3 (nLig3) or DNA polymerase theta (Polθ), two key MMEJ factors, did not affect zebrafish development but sensitized embryos to ionizing radiations and that deficiency of Polθ, but not nLig3, profoundly alters the mutation spectrum induced during repair of Cas9-mediated DSBs. By contrast, inactivation of DNA ligase 4, required for cNHEJ, did not seem to sensitize embryos to ionizing radiations nor to affect repair of Cas9-mediated DSBs but resulted in important larval growth defects. Our study underscores the dynamic and context-dependent roles of cNHEJ and MMEJ pathways during zebrafish development, highlighting their differential requirements across developmental stages and in response to genotoxic stress.

**Supplementary Information:**

The online version contains supplementary material available at 10.1038/s41598-025-88564-6.

## Introduction

DNA double-strand breaks (DSBs) are one of the most deleterious DNA lesions for the cell. They can lead to cell death if unrepaired or to mutations at the site of the break, contributing to senescence or oncogenesis, if misrepaired. To counteract these effects, living organisms have evolved several DSB repair pathways. Homologous Recombination (HR) is primarily non-mutagenic because it uses sequence homology with the sister chromatid to repair the break^1^. Canonical Non-Homologous End-Joining (cNHEJ) mostly repairs DSBs faithfully but can induce small insertions and deletions^2^. In contrast, Microhomology-Mediated End-Joining (MMEJ) is always mutagenic. MMEJ relies on short-range resection by the Mre11-Rad50-Nbs1 (MRN) complex to anneal short (2–20 bp) sequence microhomologies (MHs) on either side of the break to repair, resulting in the deletion of one of the MHs and the intervening sequence^3^. MMEJ was originally described as a backup mechanism used when HR and cNHEJ are not functional^4,5^. However, in recent years, it has been shown that MMEJ is a prominent DSB repair pathway in numerous models, even when HR and cNHEJ are active^5–12^. MMEJ has also been linked to chromosomal rearrangements and the fusion of uncapped telomeres in some cancers^13–16^.

A key factor of MMEJ is DNA polymerase theta (Polθ), encoded by the *polq* gene^17^. It is a low fidelity DNA polymerase^18,19^ responsible for multiple steps of the MMEJ pathway, including displacing Replication Protein A (RPA) from the single-stranded DNA resulting from resection and performing gap-filling synthesis^20,21^. Overexpression of this gene correlates with poor prognosis in several types of cancers^22–24^. In zebrafish, *polq* has been shown to be essential during early stages of development for survival following DNA damage induced by ionizing radiation or DSBs mediated by CRISPR/Cas9^10^. Another central factor of the MMEJ pathway is DNA ligase 3 encoded by the *lig3* gene, which is considered to be the major DNA ligase in MMEJ^25^. The *lig3* gene is essential for cell survival due to its additional function in mitochondrial DNA maintenance^26–29^. In zebrafish embryos, it has been shown using an exogenous MMEJ fluorescent reporter system and morpholino-mediated knockdown of *lig3* that MMEJ depends on Lig3^8^.

To examine the relative importance of MMEJ and cNHEJ during zebrafish embryonic development, we analyzed the expression of genes related to these pathways in the zebrafish embryo and measured the transcriptional response of these genes following genotoxic stress induced by ionizing irradiation. We also generated zebrafish mutant lines for *polq*,* lig3* and *lig4* and studied their resistance to genotoxic stress. Finally, we used DNA deep sequencing to compare the mutation spectrum induced by Cas9-mediated DSBs at several loci in *polq*,* lig3* and *lig4* mutants and wildtype embryos.

## Materials and methods

### Animals

Zebrafish (*Danio rerio*) were bred in our facility and maintained according to standard procedure^30^. Wildtype animals were Tübingen from EZRC (https://www.ezrc.kit.edu). All mutant lines were produced and kept in this background. Zebrafish embryos were obtained by natural spawning. Embryos were collected from breeding tanks and washed with E3 embryo medium. Embryos were raised at 28.5 °C in E3 embryo medium until 5 dpf. Between 5 and 15 dpf, larvae were raised at 28.5 °C in water with 5 g/L of sea salt (Instant Ocean) and fed *ad libitum* with rotifers. From 15 dpf onward, fish were kept at 26 °C on 14–10 h light-dark cycle.

### Euthanasia

In accordance with the European Union Directive (2010/63/EU), animals were euthanized using anesthetic overdose. Animals were placed in a solution of tricaine methanesulfonate (MS222, 0.03%, pH7, 26 °C) for 15 min following cessation of opercular movement.

### In situ hybridization

To generate all the probes, primers specific to each gene were designed to amplify between 800 and 1200 pb of cDNA (Primer sequences are listed in Table S1). PCR were performed on a library of cDNA made from total RNA extracted from wildtype embryos at 48 hpf. PCR products were purified on agarose gel using the Wizard SV gel and PCR clean-up system (Promega) and cloned into pGEMT-easy (Promega). Plasmid sequences were confirmed by Sanger sequencing. Sense and antisense RNA probes were synthesized with SP6 or T7 RNA polymerase by using Digoxigenin (Dig)-RNA labelling mix (Roche). In situ hybridization (ISH) experiments were carried out as previously described^31^.

### Real-time quantitative RT-PCR

Total RNA samples were purified from wildtype embryos collected at the appropriate stage, and from tissues including brain, spinal cord, eye, skin, gills, swim bladder, heart, muscle, intestine, kidney, liver, ovary, and testis dissected from adults. Embryo samples were lysed manually with a pestle in a 1.5mL test tube, and dissected adult samples were lysed using a TissueLyser II system (Qiagen), both with RNAble solution (Eurobio). Samples were treated with DNAse I (Roche) to remove potential contamination by genomic DNA and then purified with phenol/chloroform extraction. The integrity of RNA was then assayed by electrophoresis on a 1% agarose gel. cDNA samples were obtained from 2 µg of purified total RNA using Goscript reverse transcriptase (Promega) with random primers (Promega). Finally, specific cDNAs were quantified by real-time PCR using PowerUp SYBR Green (Applied Biosystem) and a QuantStudio 6 Flex instrument (Applied Biosystem). For all RT-qPCR, *lsm12B* and *mob4* were used as housekeeping genes^32^. Primer sequences are listed in Table S1. Prior to be used in qPCR, all primers pairs were assayed on 4 serial dilutions of corresponding cDNA. Linearity of CT variation on dilutions and single peak of melting curved were verified. Relative quantities were calculated using the 2e-ΔCT formula.

### Analysis of gene expression using existing zebrafish datasets

Transcriptomic data from zebrafish early embryos (0 to 5.5 hpf) were obtained from supplementary tables S3 and S4 in the publication by Bhat et al.^33^. Single-cell data from zebrafish embryos (between 3 hpf and 5 dpf) were obtained as processed Seurat object from https://daniocell.nichd.nih.gov/^34^ and analyzed using Seurat v5^35^. The dataset was first subdivided into datasets corresponding different stages, with 6 hpf corresponding to groups 3–4, 5–6 and 7–9 hpf; 14 hpf to 10–12 and 14–21 hpf; 1 dpf to 24–34 hpf; 2 dpf to 48–58 hpf; 3 dpf to 72–82 hpf; 4 dpf to 96–106 and 5 dpf to 120 hpf. Then, average expression across tissue was obtained using the DotPlot function. To calculate the proportion of cells in the G2/S/M or G1/G0 phases, we utilized the CellCycleScoring function. The reference list of cell cycle genes, originally based on human gene names, was modified to correspond to zebrafish nomenclature. For each stage, scores were calculated for all cells (average score per stage) as well as for cells expressing each selected DNA repair gene. Single-cell data from adult testis were obtained from https://github.com/asposato/aging_zebrafish_testis^36^ and average expression across cell type was analyzed as above. The analysis of gene expression in ovary^37^ was done directly on the web portal https://singlecell.broadinstitute.org/single_cell/study/SCP928.

### Ionizing radiation treatment

Embryos of selected genotypes were placed at 4 hpf or 24 hpf by group of 20 in 60 mm Petri dishes in E3 medium and submitted to Gamma irradiation under a ^137^Cs source delivering 1 Gy/min (Biobeam 8000, Institut Cochin, Paris). Following irradiation, embryos were raised up to the appropriate stage and used for RNA extraction, phenotype scoring or acridine orange staining.

### CRISPR-Cas9 guide RNA design and injection

Guide RNAs (sequences in Table S1) were designed using CRISPOR (http://crispor.tefor.net/)^38^. For the establishment of mutant lines, gRNAs were designed as described in Fig. 4. Specific crRNAs and tracrRNA were ordered from Integrated DNA Technologies and kept as a 100 µM solution in duplex buffer (Integrated DNA Technologies) at -80 °C. According to manufacturer instructions, gRNAs were obtained by producing crRNA-tracrRNA duplexes. Equal amounts of crRNA and tracrRNA were mixed and heated to 95 °C for 5 min and then allowed to cool down to room temperature. The duplex solution was then diluted at final concentrations of 20 µM of duplex in a solution of 30 mM HEPES-NaOH and 225 mM KCl. To prepare injection mixes, duplex RNAs were individually mixed with Cas9 protein to form ribonucleoprotein complexes (gRNA at 13.3 µM and Cas9 at 10 µM) and incubated at 37 °C for 10 min, then kept on ice until injection. At this stage, ribonucleoprotein solutions were mixed if required. gRNA-Cas9 complexes were injected (2 nl) into 1–2 cell-stage embryos.

To generate mutant alleles for *lig3*,* lig4* and *polq*, injected embryos were raised to adulthood and these founders were crossed against wildtype fish to screen for their ability to transmit a deleted / mutant allele by genotyping their progeny. F1 from identified founders were raised to establish the different mutant lines.

## Genotyping

For genotyping, genomic DNA was extracted from either fin clips (larvae and adult) or whole embryos in 50 mM NaOH for 15 min at 95 °C. After buffer neutralization with 1/10 vol of Tris 10 mM pH 8, the DNA solution was directly used for PCR amplification (primer in table S1). The *polq* allele is a deletion 220 pb; the *lig4* allele is a deletion of 3.3 kb corresponding to the entire unique coding exon. The *lig3* allele is a deletion of only 9 pb that misses an EciI restriction site.

***lig4*mutant growth monitoring**.

6 dpf larvae of a clutch from a cross between *lig4*^*+/−*^ were placed individually into numbered cell culture flasks (40 mL) and raised up to 28 dpf. Larvae were imaged on an Olympus SZX12 stereomicroscope at 6 dpf, 10 dpf, 14 dpf, 21 dpf and 28 dpf without knowing their genotype. At 28 dpf, larvae were euthanized and genotyped. Animal sizes were measured on pictures using ImageJ^39^.

## Acridine orange staining

To mark dead cells, we used a protocol adapted from^40^. 48 hpf embryos were dechorionated manually and placed in a 2 µg/mL solution of acridine orange (Sigma A1301) in E3 medium for 30 min. Embryos were then washed repeatedly in E3 medium before being transferred to clean E3 medium in a Petri dish. They were then imaged on a Leica DM5500B microscope. Positive cells were manually counted on pictures using ImageJ. Stained cells were counted on the tail, from the cloaca to the tip of the fin tail. Counts were normalized over the counted area.

### Sequence analysis following CRISPR/Cas9-mediated mutagenesis

Injection mixes of gRNA-Cas9 complexes were prepared as described above except that a Cas9-eGFP fusion protein was used. Ten different guides were used, pooled in two different mixes (#1, #2. five guides in each mix, sequences in Table S1). Injection mixes were prepared and injected (2 nl) independently into 1–2 cell-stage embryos. At 8 hpf, GFP-positive embryos were sorted on a Leica DM5500B microscope. At 9 hpf, wildtype, *MZpolq* and *MZlig3* embryos were pooled (20 per genotype) and genomic DNA was extracted using Wizard Genomic DNA purification kit (Promega). For *lig4* mutant embryos, heterozygous adults were mated producing 25% of *lig4* mutants. Embryos were genotyped individually, and DNA samples were pooled according to the genotype (20 embryos per pool). For each genotype (wildtype, *MZpolq* and *MZlig3*, *lig4)*, and for each injection mix (#1 and #2) DNA samples were then used for PCR amplification for each of 10 targeted loci using specific primers (see Table S1) and PCR products were purified on a 0.8% agarose gel using E.Z.N.A. Gel extraction kit (Omega Bio-Tek). Note that one locus in mix #2 could not be amplified. Amplicons were then pooled per genotype (pool 1 including 5 amplicons and pool 2, 4 amplicons.). For sequencing, Illumina adapters and index (one index per genotype) were added to amplicon pools by ligation and DNA samples were sequenced on an Illumina MiSeq (paired-end sequencing 2 × 250 bp).

For each pool, assembled reads (using FLASH^41^ version 1.2.11) were finally demultiplexed to recover individual amplicon using a custom script which looks for a 15 nt sequence of the WT amplicon at a distance of 56–57 nt upstream the cleavage site (called barcodes in Table S2).

To identify the different types of mutations, we used a custom python pipeline to count and classify insertions and deletions (InDels) in different types: 1-bp deletions, > 1-bp deletions without microhomology (MH), > 1-bp deletions associated with MH motifs whose length is at least 2 base pairs, 1-bp insertions, > 1-bp insertions and substitutions. Events were quantified in a window of 9 to 11 bp around the cleavage site to minimize false positive InDels due to sequencing errors, and comparison between treated and control samples was used to call InDels due to treatment vs. sequencing errors.

### Statistics and figure preparation

Quantitative data were analyzed using R software^42^ and the tidyverse^43^ package. Plots were made with the ggplot2^44^ package. Figures were prepared using Scribus software (www.scribus.net/).

### Ethics approval

All procedures were approved by the Institutional Ethics Committee Cuvier at the Muséum national d’Histoire naturelle (APAFIS#10180). In accordance with the European Union Directive (2010/63/EU), all efforts were made to minimize the number of animals used and their suffering. The study is reported in accordance with ARRIVE guidelines.

## Results

**Analysis of expression of MMEJ- and cNHEJ-related genes during embryogenesis.**

**Fig. 1:**
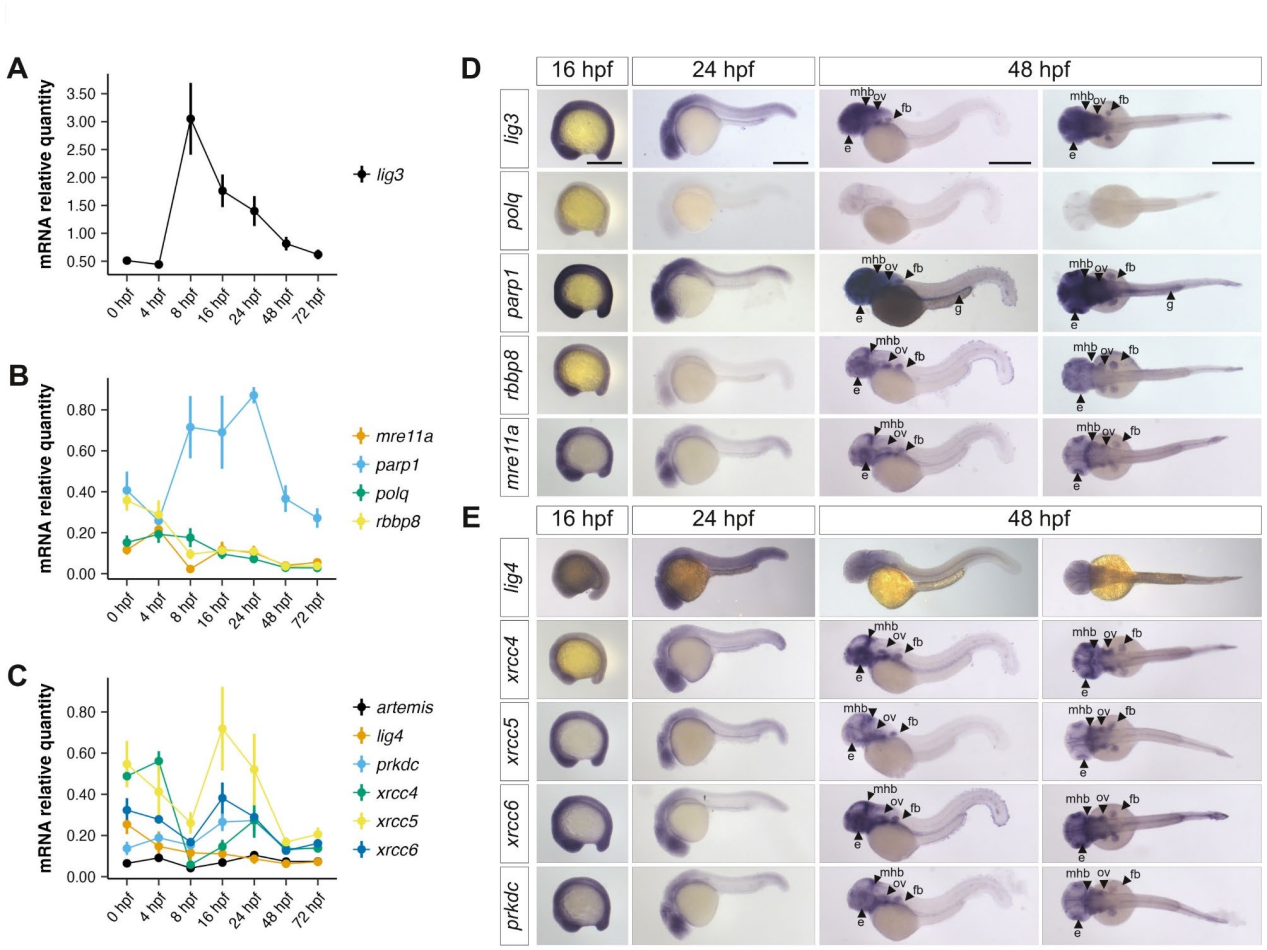
Analysis of MMEJ- and cNHEJ-related transcripts levels and distribution throughout embryonic development. (**A-C**) RT-qPCR analysis in embryos from 0 hpf to 72 hpf of relative mRNA levels for genes related to the MMEJ pathway and resection (**A-B**) and for genes related to the cNHEJ pathway (**C**). Note that, due to high expression levels, *lig3* is presented separately (**A**). Expression levels are expressed as ratios to housekeeping genes (see methods). Data are means from four independent experiments. Error-bars are s.e.m. (**D-E**) Whole mount in situ hybridization for the same genes as in (**A-C**) genes related to the MMEJ pathway and regulation of resection (**D**) or related to the cNHEJ pathway (**E**) in wildtype embryos. Note that we could not produce a probe for *artemis*. Lateral view presented for 16 hpf, 24 hpf and left column of 48 hpf. Dorsal view presented in right column of 48 hpf. fb: fin buds; g: gut; e: eye; ov: otic vesicle; mhb: midbrain-hindbrain boundary. Scale bars: 500 microns.

To assess the availability of MMEJ and cNHEJ during zebrafish embryogenesis, we first performed RT-qPCRs to analyze the expression of a selection of genes involved in each pathway at seven stages from the 1 cell-stage (0 hpf) to the early larval-stage (72 hpf) (Fig. 1A-C). All tested genes exhibited changes in expression levels during embryogenesis. Most mRNAs of MMEJ-related genes were detected at their highest levels at 0 hpf, suggesting a relatively high level of maternal contribution that gradually decreased as embryonic development progressed (Fig. 1B). Two notable exceptions were *parp1* and *lig3* that showed increased expression levels after zygotic genome activation, which occurs at 3 hpf in zebrafish. Expression peaked at 8 hpf for *lig3* (Fig. 1A) and between 8 and 24 hpf for *parp1* (Fig. 1B). For cNHEJ-related genes, overall, expression levels decreased from 0 to 8 hpf. Then, for most of them, there was an increase in expression levels up to 16–24 hpf, a period corresponding to somitogenesis and early neurogenesis, followed by a decrease between 24 and 48 hpf (Fig. 1C). One exception was *lig4* which steadily decreased from fertilization to 72 hpf (Fig. 1C). Our results suggested that all genes are maternally expressed. To confirm this, we analyzed data from a recent study that characterized maternal and zygotic contribution to gene expression during early zebrafish development (0 to 5.5 hpf) using SLAMseq^33^. Consistent with our findings, all selected genes were found to be maternally expressed. In the latter studies, over the period studied, the expression level of the DNA DSB repair genes examined appeared relatively constant except for *xrcc5* whose expression decreased (Fig. S1A-B), consistent with our own experimental results. Of note, the analysis of zygotic contributions (when available) suggests that these genes become actively transcribed between 4.5 and 5 hpf (Fig. S1A-B).

To gain insight into potential tissue-specific activity of MMEJ or NHEJ, we then used in situ hybridization (ISH) to analyze the expression of these genes during embryogenesis, on embryos at 16, 24 and 48 h post fertilization (hpf). At 16 hpf, all analyzed genes showed ubiquitous expression except for *polq* whose transcripts could not be detected (Fig. 1D). At 24 hpf, expression patterns began to be more regionalized with most genes being more strongly expressed in the developing brain, including the eyes, and spinal cord (Fig. 1D-E). At this stage, as well as at 48 hpf, *polq* mRNA remained undetectable (Fig. 1D). By 48 hpf, all studied genes, except *lig4*, showed regionalized expression. *lig3* and *parp1* were strongly expressed in the head with some expression in the pectoral fin buds, and the developing gut for *parp1* (Fig. 1D-E). The other genes shared a similar regionalization pattern, being primarily detected in the eyes and the midbrain-hindbrain boundary (MHB) as well as in the fin buds and otic vesicles (Fig. 1D-E). Thus, the majority of cNHEJ- and MMEJ-related genes analyzed presented regionalized expression during embryonic development, with higher expression in the developing brain at 24 hpf, and in the eye and MHB at 48 hpf (Fig. 1). These correspond to regions with cell proliferation^45–47^, suggesting an enrichment of DNA repair gene expression in actively dividing tissues.

**Fig. 2:**
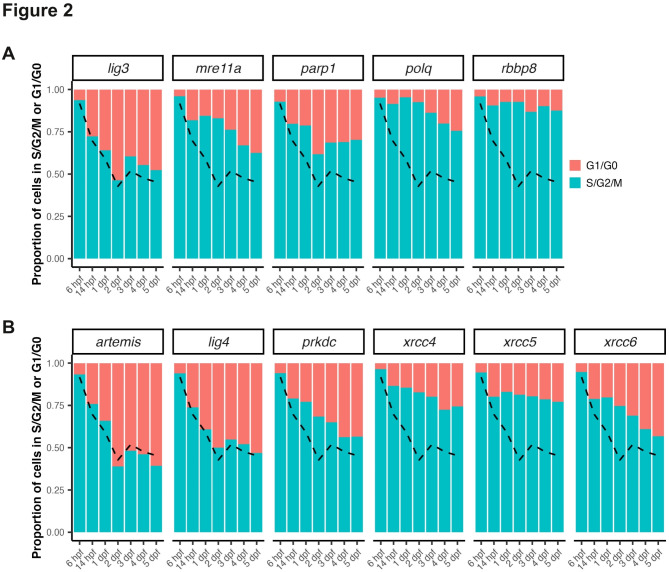
Analysis of mitotic activity in cells expressing MMEJ- and cNHEJ- related genes during embryogenesis. (**A**-**B**) Proportion of cells in S, G2 or M phase in cells expression genes related to MMEJ and resection (**A**) or related to cNHEJ (**B**) according to stage during embryonic development. In all panels, the dashed line indicates the average value for all cells at the corresponding stage. Data were computed from single-cell transcriptomic data from zebrafish embryo between 3 hpf and 5 dpf published by Sur et al.^34^ (see methods).

To further analyze the expression of this selection of DNA repair genes, we took advantage of available single-cell RNA sequencing data from zebrafish embryos^34^ (see methods). Looking at the gene expression across tissues at 14 hpf, and 1, 2 and 5 dpf showed that several of these genes where more highly expressed in the endoderm and the gut (Fig. S2). This was notably the case of *parp1*, consistent with our ISH results. Of note, these data also revealed that most genes where enriched in primordial germ cells (PGC)(Fig. S2). Next, since our ISH results suggested that many tested genes were enriched in actively dividing tissues, we used this single-cell dataset to compute, for different stages, the proportion of cells in S, G2 or M phase; or in G1 or G0 phase among cell expressing each of selected of cNHEJ- and MMEJ-related genes, and all cells (Fig. 2. see method). As expected, the global proportion of cells in S/G2/M, corresponding to actively dividing cells, progressively decreased as development progressed (Fig. 2. see dashed line). However, for cells expressing most cNHEJ- and MMEJ-related genes, the proportion of actively dividing cells remained much higher (Fig. 2). Interestingly, the two genes with the highest proportion of cell in S/G2/M were *polq* and *rbbp8*, both MMEJ-related genes (Fig. 2).

Together, these results showed that the expression levels of the majority of cNHEJ- and MMEJ-related genes assayed are dynamic during embryonic development and become regionalized enriched in actively dividing tissue tissues as development progresses.

### Expression of MMEJ- and cNHEJ-related genes in adult tissues

**Fig. 3:**
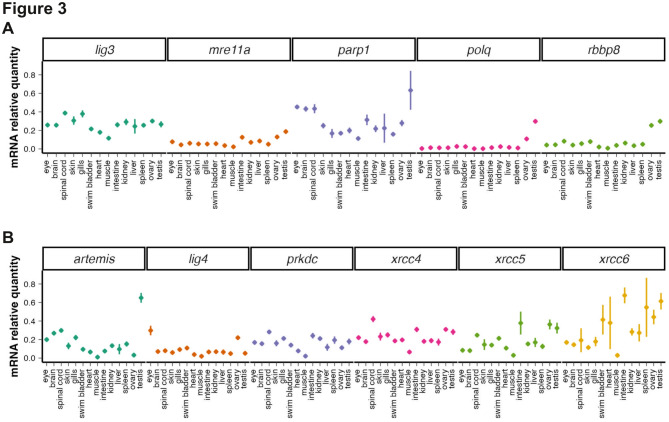
Analysis of mRNA levels of MMEJ- and cNHEJ- related genes across adult tissues. (**A-B**) RT-qPCR analysis for genes related to the MMEJ pathway and resection (A) and for genes related to the cNHEJ pathway (**B**) in adult fish tissue. Expression levels are expressed as ratios to housekeeping genes. Data are means from four independent experiments. Error-bars are s.e.m.

To assess the availability of MMEJ and cNHEJ in adult tissues, we dissected fish and conducted RT-qPCR analysis on the same genes as above. Strikingly, all genes showed differential expression between tissues. Similarly to what was observed with ISH, many of these genes were highly expressed in the brain and spinal cord (*lig3*,* parp1*,* artemis*,* prkdc*,* xrcc4*,* xrcc5*,* xrcc6*), as well as in the intestine (*lig3*,* parp1*,* prkdc*,* xrcc4*,* xrcc5*,* xrcc6*). Consistent with the fact that their gene products form a key multiprotein complex in NHEJ, *prkdc*, *xrcc4*,* xrcc5* and *xrcc6* exhibited similar expression profiles (Fig. 3B). Interestingly, the lowest levels of expression for all the studied genes were found in skeletal muscle (Fig. 3A-B). Additionally, all genes were detected at relatively high levels in the gonads. In particular, *polq* and *rbbp8*, were strongly expressed in the gonads compared to other tissues (Fig. 3A). While many genes presented similar expression levels in testis and ovary, some genes also showed higher expression in one sex (*parp1*,* polq* and *artemis* expression levels were higher in testis and *lig4* in the ovaries). We then analyzed published single-cell transcriptomic data from zebrafish testis^36^ an ovaries^37^. While expression levels could not be directly compared between the two datasets, no obvious difference was found between the two gonads (Fig. S3). In contrast, a similar pattern was observed, with most genes undetected in somatic cells, but expressed in the germ cell lineage (Fig. S3). This observation aligns with embryonic data suggesting expression in PGC. Besides, genes showed relatively high expression levels prior and during meiosis but much lower in differentiating gametes (Fig. S3). In summary, all examined DNA DSB repair genes were differentially expressed between tissues and were highly detected in the gonads, most likely due to expression in the germ cell lineage.

### Ionizing radiation exposure increases the expression of CNHEJ- and MMEJ-related genes at 24 hpf but not at 4 hpf

Upregulation of DNA repair genes is a common transcriptional response to genotoxic stress, often mediated by Tp53^48^. We therefore chose to examine whether exposure to ionizing radiation (IR) during embryonic development would induce a differential or common transcriptional responses in cNHEJ- and MMEJ-related genes. Embryos were subjected to a 6 Gy dose of IR at 4 hpf, when cells are rapidly dividing, and at 24 hpf when development is more advanced, and embryos contain more post-mitotic cells. Two hours after irradiation, RNAs were extracted, and RT-qPCRs were performed.

**Fig. 4:**
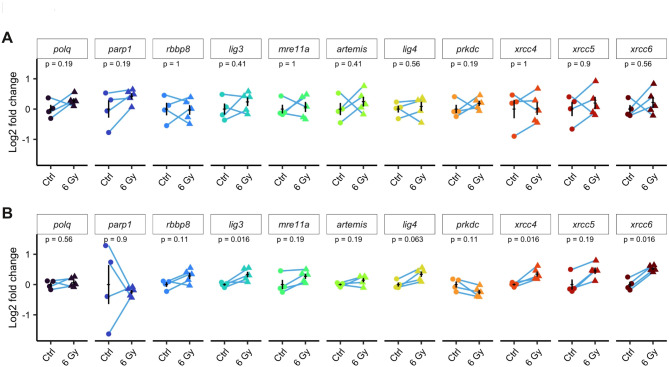
Analysis of mRNA levels of MMEJ- and cNHEJ-related genes in response to ionizing radiation exposure in embryos. (**A-B**) Embryos were exposed to a 6 Gy dose of ionizing radiation either at 4 hpf (**A**) or at 24 hpf (**B**). They were then left to develop for 2 h before mRNA extraction. Samples were pools of 40 (4 hpf) or 20 (24 hpf) embryos. Blue lines indicate samples from the same clutch of eggs (*N* = 4 for control and *N* = 5 for irradiated). Data are presented as Log2 of fold change compared to controls. Error-bars are s.e.m. p values from Wilcoxon tests.

Our results showed that at 4 hpf, although the zygotic genome is already transcriptionally active at this stage^49^, none of the studied genes exhibited significant changes in expression in response to IR (Fig. 4A). It is noteworthy that at this stage, mRNA levels detected by RT-qPCR were relatively variable in both control and irradiated embryos. In contrast, at 24 hpf, the expression level of many DNA DSB repair genes increased in response to IR. Specifically, *lig3*, *xrcc4*, and *xrcc6* showed significant increases in mRNA levels in irradiated embryos compared to untreated controls (Fig. 4B). In addition, although not statistically significant, *lig4*, *mre11*,* rbbp8*, and *xrcc5* showed a clear tendency towards increased expression. Interestingly, *prkdc* mRNA levels seemed reduced in irradiated embryos, although this difference was not statistically significant. Lastly, IR exposure did not affect *polq* expression levels.

These results suggest that while during the first hours of development, embryos do not seem to transcriptionally respond to IR, at 24 hpf there is an adaptative transcriptional response to DNA damage induced by IR involving both MMEJ- and cNHEJ-related genes.

### Establishment of MMEJ- and cNHEJ- deficient zebrafish lines

**Fig. 5:**
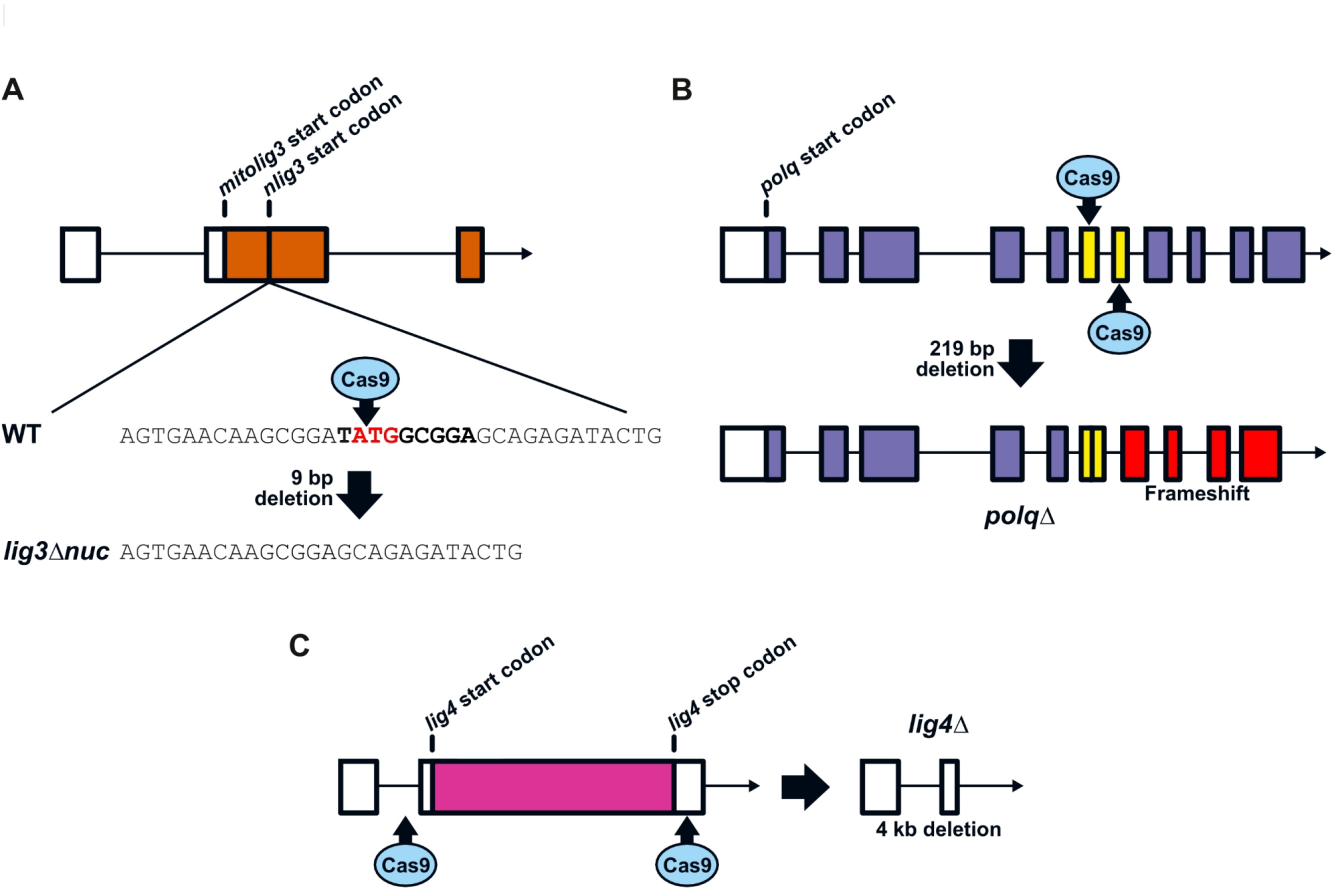
Inactivation of MMEJ and cNHEJ DNA repair pathways through CRISPR/Cas9-mediated gene knockout. (**A-C**) Schematics depicting the different gene knockout strategies used to inactivate the MMEJ and cNHEJ pathways. (**A**) Inactivation of the nuclear isoform of DNA ligase 3 (nLig3) was performed by inducing a small in-frame deletion of the nuclear isoform’s start codon. (**B**) The *polq* gene coding for DNA polymerase theta (Polθ) was knocked out by inducing a large deletion causing a frameshift early in the open reading frame. (**C**) The *lig4* gene coding for DNA ligase 4 was knocked out by inducing a large deletion of the coding sequence by targeting non-coding DNA on either side.

To functionally test and compare the importance of MMEJ and cNHEJ during zebrafish embryonic development, we established mutant lines for three genes: *lig3* and *polq* to inhibit MMEJ, and *lig4* for cNHEJ. *Lig3* encodes a DNA ligase required for MMEJ^25^. The *lig3* gene encodes both a nuclear (hereafter referred to as nLig3) and a mitochondrial isoform of the protein, the latter being essential for cell survival^26–29^. The two isoforms of the protein are translated from different start codons on the transcript, with the mitochondrial start codon located 5’ to the nuclear start codon (Fig. 5A). We, therefore, set to specifically knock-out the nuclear isoform by deleting the corresponding start codon using CRISPR/Cas9. We obtained fish that transmitted a 9 bp deletion removing the nuclear start codon and preventing the production of nLig3 while preserving mitochondrial Lig3.

For *polq*, we used two guide RNAs to induce a deletion between exons 6 and 7 (out of 32) and screened founder fish for a frameshift-inducing deletion. We obtained F0 fish transmitting a 219 bp deletion resulting in a frameshift causing a premature stop codon at position 397 of the mutant protein (full-length Polθ is 2576 aa) (Fig. 5B).

To create the *lig4* mutant line, we initially used two guide RNAs targeting the single coding exon of this gene. However, all injected larvae displayed strong growth defects and could not be raised to adulthood. DNA sequencing of individual larvae revealed frame-disrupting mutations on both alleles, likely causing Lig4 inactivation and the observed phenotype. Since *lig4* comprises only one coding exon, we decided to delete it entirely by using guide RNAs targeting non-coding regions on both sides of the coding sequence. We reasoned that these guide RNAs would not induce deleterious mutations independently and that the limited rate of deletion would not prevent normal growth (Fig. 5C). With this approach, injected animals reached adulthood and we were able to identify founders transmitting the desired deletion.

For each gene targeted, one founder was then crossed with wildtype fish to produce F1 offspring and establish the mutant line. In all three cases, heterozygous fish were viable and fertile without any apparent defect.

### Polθ and nLig3 are dispensable, but Lig4 is required for larval growth

We produced homozygous mutants for *polq*,* lig3*, and *lig4* to assess their role in DNA repair during development. Fish homozygous mutant for *lig3* and *polq* developed without visible defects and adults were viable and fertile (not shown). To test for the possibility of an embryonic requirement masked by maternal mRNAs or proteins deposited in the egg during oocyte maturation, we used homozygous females to produce maternal-zygotic (MZ) mutants. These *MZpolq* and *MZlig3* mutants, which were homozygous for the mutation and lacked respectively *polq* or *lig3* transcripts or protein in the egg, developed normally and adults were viable and fertile (not shown). These results suggested that both Polθ and nLig3 are dispensable for normal development and growth.

**Fig. 6:**
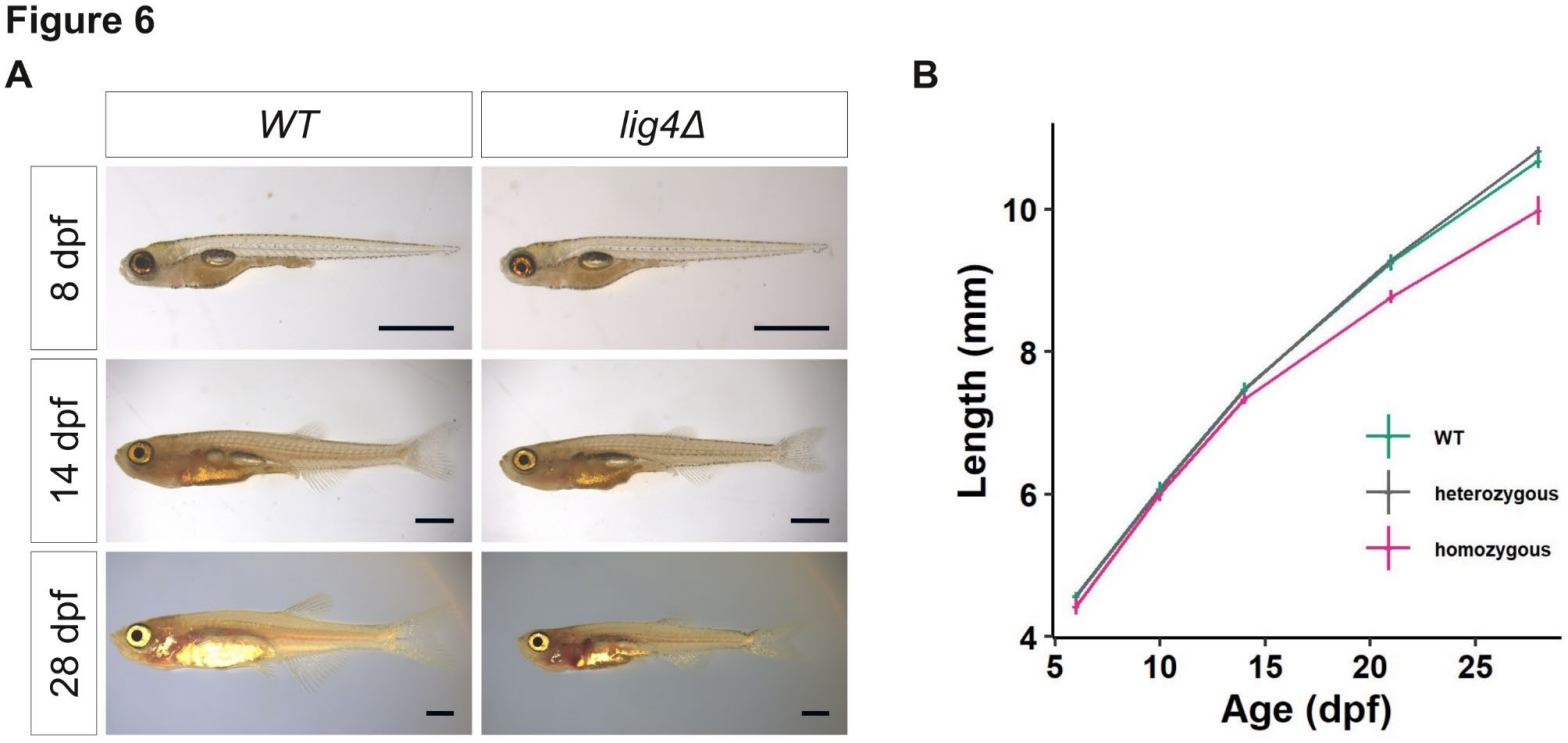
DNA ligase 4 is required for larval growth. (**A**) Comparison of wildtype (WT) and *lig4* mutant sibling larvae development from 8 dpf to 28 dpf, same fish in the three pictures on each column, scale bar = 1 mm. (**B**) Growth monitoring of sibling larvae from a *lig4* heterozygous cross (*n* = 8 homozygous mutants, *n* = 13 heterozygous, *n* = 9 wildtype), from 8 dpf to 28 dpf, error-bars are s.e.m.

In contrast, approximately a quarter of the larvae from incrosses between *lig4*^+/−^ exhibited significant growth defects and failed to reach adulthood, requiring euthanasia. Post-mortem genotyping confirmed that these larvae were homozygous mutants for *lig4*. To characterize this growth defect, sibling larvae were raised individually, in numbered flasks, to prevent competition for food with non-homozygous mutant embryos, which could artificially exacerbate the phenotype. Fish were imaged at 6, 10, 14, 21, and 28 dpf, blindly with respect to their genotype. All larvae were euthanized at 28 dpf and genotyped. Our analysis revealed that *lig4* heterozygous mutants grew at the same rate as wild-type siblings (Fig. 6B) and showed no defects. In contrast, *lig4* homozygous mutants grew normally until 14 dpf but appeared smaller than their siblings thereafter (Fig. 6B). By 28 dpf, *lig4* homozygous mutants exhibited misshapen heads and abdomens (Fig. 6A).

Thus, while Polθ and nLig3 seemed dispensable for normal development and growth, Lig4 was essential for zebrafish larval growth. Consequently, for subsequent experiments, homozygous *lig4* mutant embryos could only be obtained from crosses of heterozygous *lig4* mutant fish, meaning that early stage *lig4* mutant embryos contained wildtype *lig4* mRNAs of maternal origin.

### nLig3 and Polθ loss-of-function sensitize embryos to ionizing radiation, but not Lig4 loss-of-function

**Fig. 7:**
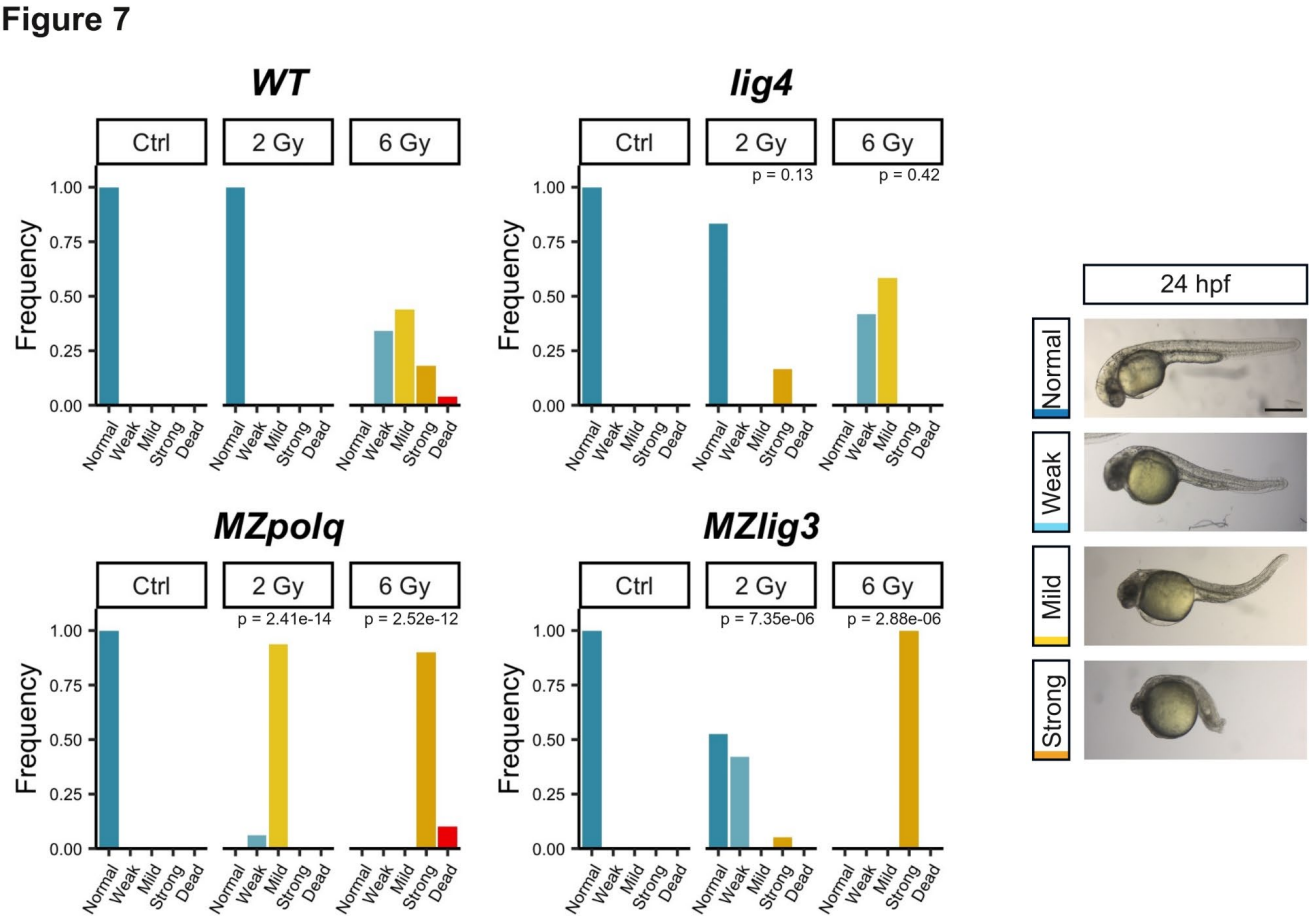
Inactivation of MMEJ sensitizes zebrafish embryos to IR. Embryos at 4 hpf were exposed to ionizing radiation (0, 2 and 6 Gy) and allowed to develop until 24 hpf. Phenotype was scored at 24 hpf according to the right panel scale. Weak = minor deformation of the tail, Mild = major deformation of the tail, Strong = atrophied tail and head, dead embryos were not imaged. WT, *n* = 140; *MZlig3*, *n* = 59; *MZpolq*, *n* = 71; and *lig4*, *n* = 22. P-values from Fisher’s Exact Test are indicated. Results for each mutant genotype at each dose were compared to those of wild-type samples at the corresponding dose. Image scale bar: 500 microns.

To test the importance of MMEJ and cNHEJ in the response to DSBs induced by genotoxic stress during embryonic development, we exposed *lig4*,* MZpolq* and *MZlig3* mutants as well as wildtype embryos to IR at 4 hpf and evaluated their phenotype at 24 hpf. At a dose of 2 Gy, wildtype embryos developed without any visible defect (Fig. 7). Most *lig4* mutants developed normally, with only 16.7% of mutant embryos showing defects at 24 hpf (Fig. 7). In contrast, 47.4% of *MZlig3* and 100% of *MZpolq* mutants exhibited embryonic defects at the same dose (Fig. 7). At 6 Gy, all wildtype embryos showed defects which we categorized in four groups based on severity. *lig4* mutants only showed weak or mild defects. However, 100% of *MZlig3* mutants and 90% of *MZpolq* mutants exhibited a strong phenotype, with the remaining 10% of *MZpolq* mutants being dead. These findings indicated that nLig3 and Polθ are required for managing DNA damage induced by IR during early embryogenesis. *lig4* mutants did not appear more sensitive than wildtype, which could be due to minimal contribution of cNHEJ to DSB repair during these early stages or to the presence of wild type *lig4* mRNAs of maternal origin at this early developmental stage.

**Fig. 8:**
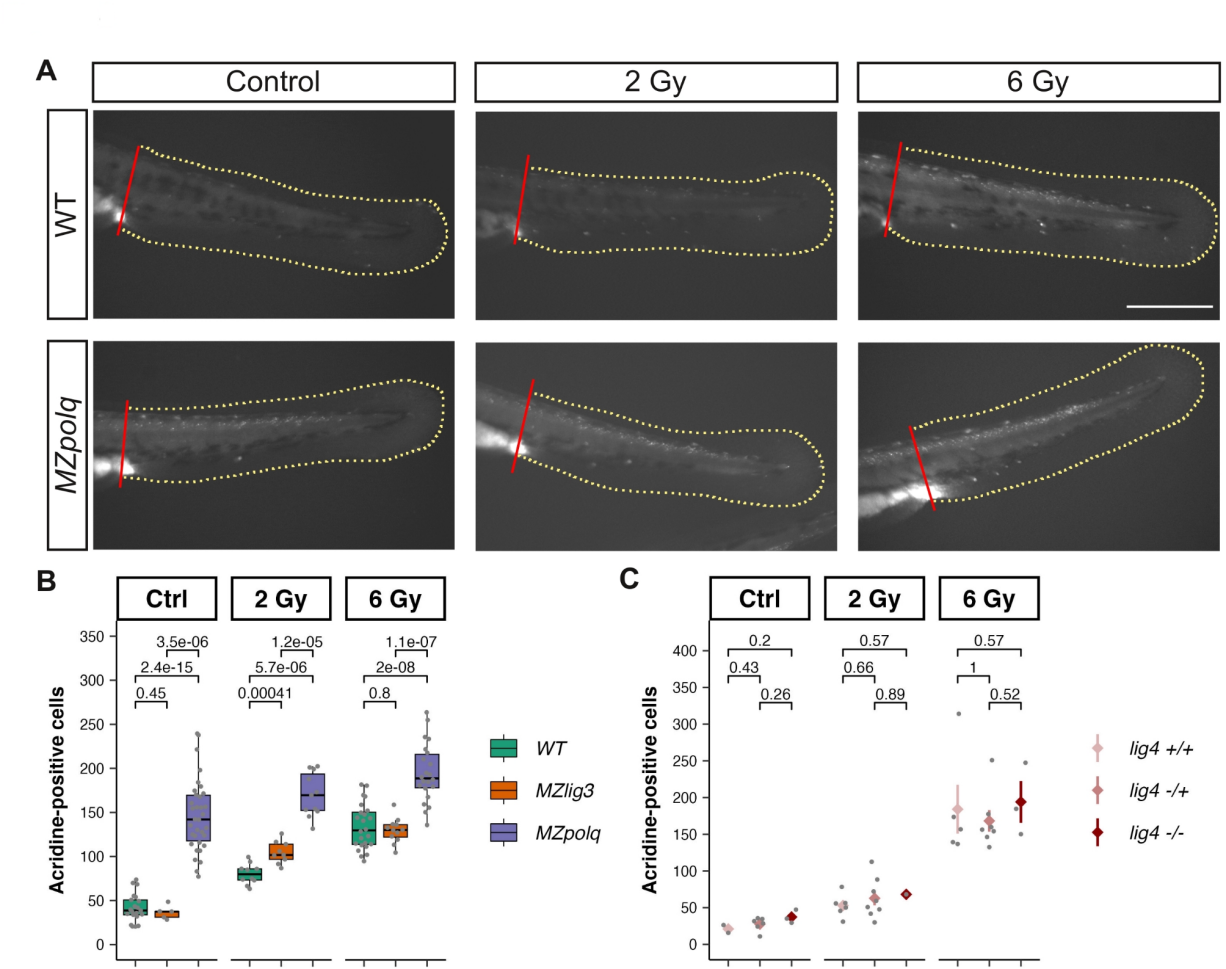
Cell death following irradiation in MMEJ and cNHEJ mutants. 24-hpf embryo from wildtype, *MZpolq* and MZ*lig*3 and from *lig4* heterozygous crosses were exposed to ionizing radiation (0, 2 and 6 Gy) and allowed to develop until 48 hpf. Cell death was then assayed using acridine orange staining (see methods) and quantified in the tail region. (**A**) Representative images of non-irradiated controls and irradiated with 2 and 6 Gy for wildtype and *MZpolq* mutant. Note the high number of fluorescent cells in MZ*polq* in both the control and the irradiated conditions. The red and the yellow lines delimit the area used for quantification. Scale bar: 200 microns. (**B-C**) Quantification of positive cells in WT, *MZpolq*, MZ*lig*3 mutants (**B**) and in *lig4* mutant (**C**). Cell numbers were normalized over the counted area. Note that *lig4* mutants were obtained from heterozygous crosses and that embryos were genotyped after staining and scoring. Results from homozygous mutants, heterozygous, and wildtype siblings are presented. p.values from Wilcoxon tests.

Next, we exposed wildtype and mutant embryos to IR at 24 hpf and evaluated their phenotypes at 48 hpf. Unlike the severe phenotypes observed in embryos irradiated at 4 hpf, neither wildtype nor mutant embryos showed any gross defect when irradiated at 24 hpf (data not shown). Nevertheless, it was likely that IR could cause cell death as a result of DNA damages. We used acridine orange to stain dead cells^50^ and we quantify positive cells in the tail (region posterior to the cloaca. See Methods). Non-irradiated wildtype control embryos exhibited basal levels of cell death (Fig. 8A, B; median = 30 acridine orange-stained cells per area unit (AU)). Upon exposure to IR, the number of dead cells in wildtype embryos increased clearly with the dose (median = 80 acridine orange-stained cells/AU at 2 Gy, and 120 cells/AU at 6 Gy, Fig. 8A, B). Strikingly, non-irradiated *MZpolq* mutants had a high number of acridine orange-stained cells (median = 140 acridine orange-stained cells/AU cells), even higher than irradiated wildtypes (Fig. 8A, B). This suggested that, although Polθ was dispensable for animals to reach adulthood without any obvious defect, it was essential for coping with DNA damages occurring during normal development. Following irradiation, *MZpolq* mutants presented increased numbers of dead cells (Fig. 8A, B). However, compared to wildtypes, this increase was only moderate (from 140 acridine orange-stained cells/AU in non-irradiated animals to 170 acridine orange-stained cells/AU at 2 Gy (*p* = 0.042, compared to non-irradiated, Wilcoxon test), and 190 acridine orange-stained cells/AU at 6 Gy (*p* = 0.053, compared to 2 Gy, Wilcoxon test).). In *MZlig3* embryos irradiated with 2 Gy, the number of dead cells was slightly increased compared to wildtypes while in non-irradiated controls and with 6 Gy, it was similar to that of wildtypes (Fig. 8B). Finally, with *lig4* mutants, since the experiment had to be done on clutches from crosses between heterozygous parents, irradiation, staining and imaging were performed prior to genotyping. We only had limited numbers of homozygous mutant embryos, but the results seemed comparable to that with wildtypes both in control and in irradiated embryos (Fig. 8C).

Our studies of zebrafish embryos’ response to irradiation suggested that Polθ and nLig3 are required to cope with IR-induced DNA damage during early stages of embryogenesis. Consistent with previous findings^10^, this supports that MMEJ is crucial for the DNA DSB repair pathway during early embryogenesis in zebrafish. In contrast, embryos were much more resistant to IR at later developmental stages. They showed no overt morphological phenotypes after irradiation at 24 hpf and inactivation of DSB repair genes did not significantly increase the apparent sensitivity of embryos to IR. Strikingly, Polθ deficiency resulted in higher number of dead cells in the trunk than irradiation at 24 hpf with 6 Gy in wildtypes.

### Polθ deficiency profoundly alters the mutation spectrum after DSB, unlike nLig3 and Lig4 deficiencies.

**Fig. 9:**
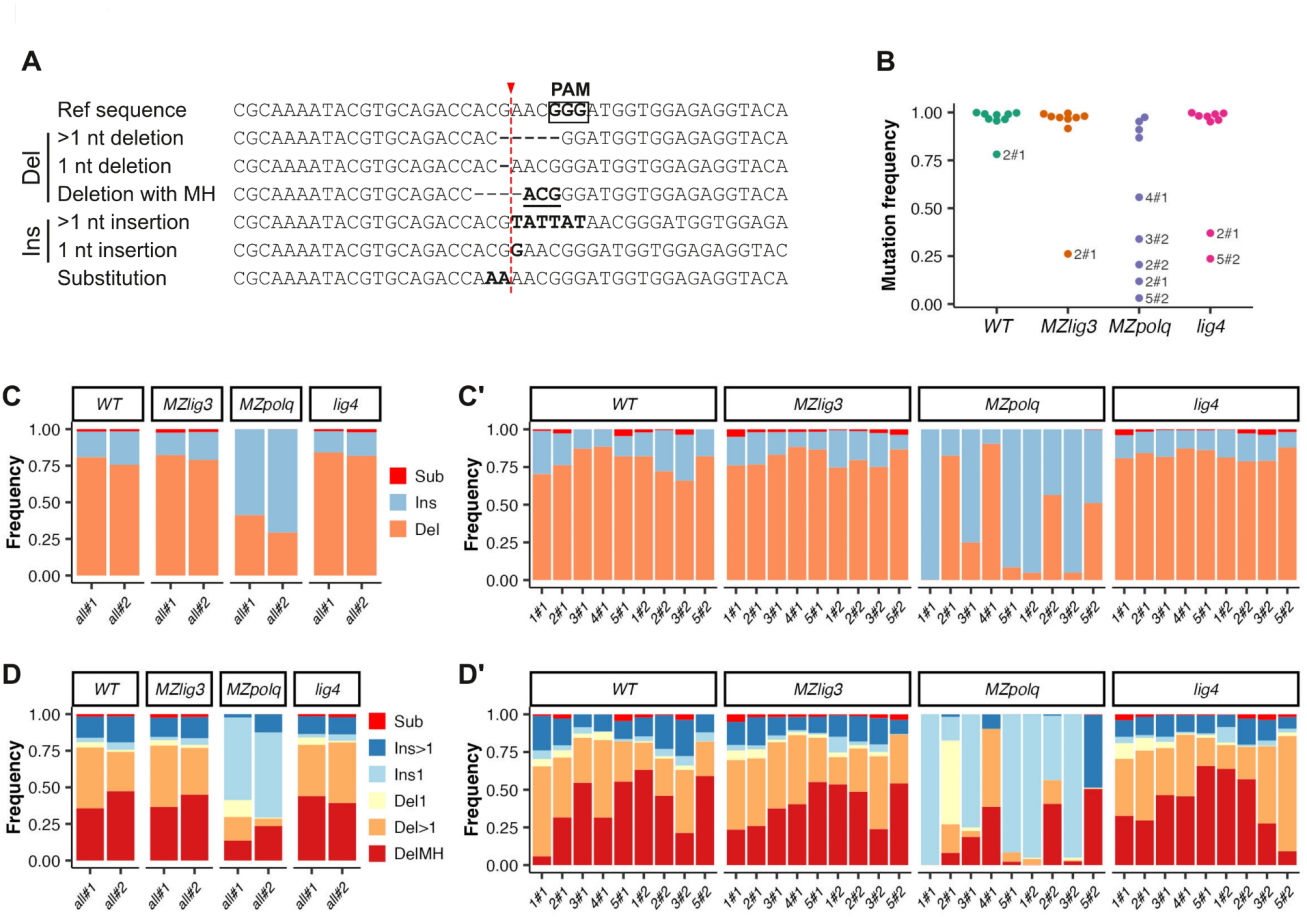
NGS analysis of the mutation spectrum after CRISPR/Cas9-mediated mutagenesis. CRISPR/Cas9-mediated mutagenesis was performed at 10 loci by injecting independently two mixes (#1 and #2) of five Cas9-GFP RNPs into zygotes (1–2 cell stage). For each mix, DNA was extracted at 9 hpf from pools of 20 embryos and PCR amplicons from all loci were sequenced, excepted one that could not be amplified. (**A**) Alignment representing examples of the different types of mutations that were quantified. In the reference sequence, the box indicates the PAM and the red dashed line the Cas9 cut site. Underlined nucleotides indicate MH. (**B**) Mutation frequency (number of mutant reads/total number of reads) for individual loci (points). (**C-C’**) Distribution of mutation types obtained globally for the two mixes (#1 and #2) (**C**) and at individual locus (**C’**) (Sub = substitutions; Ins = insertions; Del = Deletions). (**D-D’**) Distribution of mutation subtypes obtained globally (**D**) and at individual locus. (**D’**) (Sub = substitutions; Ins > 1 = insertions of 2 or more base pairs; Ins1 = insertion of 1 bp; Del > 1 = Deletions of 2 or more base pairs; Del1 = deletions of 1 bp; DelMH = deletions with MH).

DSB repair by MMEJ systematically results in small deletions flanked by microhomologies^3^. To evaluate the relative use of MMEJ and cNHEJ following DNA DSBs during embryonic development, we analyzed the mutation spectrum resulting from CRISPR/Cas9-induced DSBs in wildtype, *MZpolq*, *MZlig3*, and *lig4* mutant embryos. We designed ten guide RNAs, chosen to target genes not involved in early embryonic development (see methods). These ten guide RNAs were divided into two mixes of five and each mix injected in 1–2 cell-stage embryos. As previously reported^51^, *MZpolq* mutant embryos were highly sensitive to CRISPR/Cas9 injection and failed to develop up to 24 hpf (data not shown). Therefore, we collected embryos at 9 hpf for all genotypes. Subsequently, all target loci were amplified by PCR and amplicons were sequenced by NGS (see Methods). One locus (corresponding to one specific guide RNA, 4#2) could not be amplified and was excluded from further analysis. Sequences from amplicons were analyzed using a custom python pipeline to categorize mutations into substitutions, insertions and deletions with the latter further sorted by size and the presence of microhomologies flanking deletions (Fig. 9A).

First, our analysis revealed that in wildtype embryos, the mutagenesis was very efficient, with more than 95% of mutated sequences at all sites, except site 2#1 which had a mutation frequency of 78% (Fig. 9B). Mutation frequencies were also over 95% in *MZlig3* and *lig4* mutants at all sites except at site 2#1 (26% for *MZlig3* and 37% for *lig4* mutants) and at site 5#2 for *lig4* mutants (24%). In *MZpolq* mutants, consistent with previous studies^10^, the mutation frequency was much lower than in wildtypes at 5/9 sites, with sites 2#1 and 5#2 exhibiting only 3% and 11% of mutated sequences, respectively.

When we analyzed the types of mutations induced, we observed that in wildtype embryos, the mutation profiles were similar across all sites. The majority of detected mutations were deletions, representing 60 to 88% of mutated sequences (Fig. 9C-C’). There was also a significant proportion of insertions (11–30%) and very few substitutions (0 to 5%). In *MZlig3* and in *lig4* mutants, the results were similar (Fig. 9C-C’). However, in *MZpolq* mutants, the results were dramatically different. At most sites, the proportion of insertions increased at the expense of deletions, whose proportion was strongly reduced (Fig. 9C-C’). Interestingly, the sites where the frequency of insertions remained low were those associated with lower mutation frequencies (Fig. 9B-C’). Finally, we analyzed in more detail the mutation profiles with the quantification of cNHEJ- and MMEJ- specific signatures (Fig. 8D). Consistent with a major role for MMEJ in DSB repair during early embryogenesis, we observed that in wildtype embryos, as well as in *MZlig3* and in *lig4* mutants, a large proportion of mutations were deletions associated with microhomology, the signature of MMEJ repair^3^. Conversely, the frequency of 1-nucleotide-insertions/deletions, typically resulting from cNHEJ-mediated repair of Cas9-induced DSB breaks^52^ was very low (Fig. 9D-D’). In contrast, in *MZpolq* mutants, mutations were mostly 1-nucleotide-insertions, and a 1-nucleotide-deletion at one site, suggesting that in absence of Polθ, DSBs are repaired by cNHEJ.

In conclusion, our results showed that while the mutation spectrum following DNA DSB repair was not affected in *lig4* and *MZlig3* mutants, it was significantly altered in *MZpolq* mutants. The absence of Polθ resulted in a strong reduction of MH-flanked deletions typical of MMEJ, confirming the critical role of Polθ in this DNA DSB repair pathway.

## Discussion

In this study, we examined the relative contributions of cNHEJ and MMEJ pathways during zebrafish development by investigating the expression dynamics of their related genes under normal conditions and in response to IR, along with phenotypic characterization of mutant lines for key pathway genes. Our findings confirm prior research indicating the essential role of Polθ in surviving DSBs during early developmental stages^10^ and align with other studies highlighting the importance of MMEJ across various animal models^5–12^. We further highlight the relative importance of MMEJ and cNHEJ during zebrafish development as discussed below.

We first examined the expression profiles of MMEJ- and cNHEJ-related genes throughout zebrafish development and in response to IR. Our results revealed significant mRNA levels at 0 hpf for most MMEJ- and or cNHEJ-related genes studies (Fig. 1). Such maternal contribution of these transcripts most likely reflects the importance of DNA repair during the first hours following fertilization, a period marked by rapid cell divisions^53^. Later during development, after activation of the zygotic genome, the expression of most MMEJ- and NHEJ-related genes appeared regionalized with higher expression in specific regions. In particular, many genes were found expressed in the developing brain at 24 hpf, and in the eye and MHB at 48 hpf (Fig. 1). These correspond to cell proliferation regions^45–47^, suggesting an enrichment of DNA repair gene expression in progenitor cells. Consistent with this idea, in adults, mRNAs of most genes examined were detected at high levels in the gonads. However, our analysis did not reveal obvious differences in the dynamic or regionalization of MMEJ- compared to cNHEJ- related genes. Along the same line, we observed that, in 24 hpf embryos, IR resulted in an increased mRNA level of several genes, but there was no striking difference in the response of MMEJ- compared to cNHEJ- related genes (Fig. 4). Taken together, our studies of gene expression during zebrafish development do not highlight major sites of markedly different expression between MMEJ- and cNHEJ related genes and suggests that both pathways are deployed throughout early embryonic development, and that increased expression may take place in actively dividing cells such as progenitor cells of the MHB.

To further elucidate the functional roles of the cNHEJ and MMEJ pathways, we generated and characterized *polq*, *lig3* and *lig4* mutant zebrafish lines. At 4 hpf, Polθ-deficient embryos were considerably more sensitive to IR than wildtype embryos (Fig. 7) and MMEJ-mediated repair of Cas9-induced DSBs was drastically reduced in this background (Fig. 8). Our *polq* mutant line thus confirmed the essential role of Polθ in early embryonic DSB repair, consistent with Thyme and Schier’s previous report findings^10^. Additionally, we examined the impact of nuclear Lig3 deficiency. The proportion of MMEJ-mediated repair following CRISPR/Cas9-induced DSBs was not affected in *lig3* mutant embryos (Fig. 9) suggesting that deficiency in nuclear Lig3 did not impact MMEJ-mediated repair during early embryogenesis. This contrasts with a previous report where inhibition of Ligase 3 either by morpholino injection or using a dominant-negative form of Lig3 reduced the frequency of MMEJ repair^8^. We produced an allele designed to only block the production of the nuclear isoform while maintaining expression of the mitochondrial isoform which is needed for embryo survival. This allele is a deletion of 9 bp, that remove the translation start codon specific to the nuclear isoform of Lig3. Therefore, we cannot exclude the possibility that cryptic start codons allow the production of some nuclear Lig3. It is also possible that mtLig3 remained nuclear and compensate for the absence of the nuclear isoform. Also, nuclear ligase 3 deficiency may be compensated by other DNA ligases. Despite having different activities in DNA ligation assays performed in vitro^54^, Ligase 1 has previously been suggested to be redundant with Ligase 3 for repair of Cas9-induced DSBs in human cells^25,55^. We note, however, that zebrafish *lig3* mutant embryos were more sensitive to IR than wildtypes, suggesting that functional redundancy does not take place during repair of DNA damage inflicted by IR. IR-induced damages include DSBs but also many other types of DNA damages such as single-strand breaks and oxidized bases known to be repaired by pathways involving Ligase 3^57,58^. Like *lig3* mutants, *lig4* mutants exhibited no alteration in the mutation spectrum induced by Cas9 at any of the sites tested and did not appear more sensitive to IR than wildtype. Lig4 appears uniquely required for cNHEJ and other DNA ligases are not thought to be able to compensate for the lack of Lig4^59^. Since *lig4* mutants were not viable, we could not produce *MZlig4* mutants, and it is thus possible that maternal lig4 mRNAs (which were readily detected Fig. 1A) masked the consequence of zygotic loss-of-function during early embryogenesis. During larval growth however, *lig4* was required for normal larval growth whereas *polq* and *lig3* were dispensable.

Finally, our gene expression studies revealed highly regionalized expression patterns of numerous cNHEJ- and MMEJ-related genes, suggesting tissue-specific requirement for distinct DSB repair pathways during development. Notably, although *polq* was not essential for normal development, we observed a significant increase of dead cells in 24 hpf embryos lacking Polθ. MMEJ was recently found to be essential during mitosis of cultured cells^59^. Polθ may thus safeguard dividing cells from DSBs generated by replicative stress that persist during mitosis in rapidly dividing tissues, thereby explaining the heightened cell death in its absence. A study of DNA repair pathway mutants also recently highlighted the importance of HR genes for larval growth^60^. Further analyses of DSB repair mutants at the level of specific tissues will help to refine our understanding of the relative contribution of DSB repair pathways during larval stages of development.

In summary, our findings underscore the dynamic and context-dependent roles of cNHEJ and MMEJ pathways during zebrafish development, highlighting their differential requirements across developmental stages and in response to genotoxic stress.

## Electronic supplementary material

Below is the link to the electronic supplementary material.

Supplementary Table S1

Supplementary Table S2

Supplementary Figure S1

Supplementary Figure S2

Supplementary Material 2

## Data Availability

Sequence data that support the findings of this study have been deposited on Zenodo and are publicly accessible here 10.5281/zenodo.12820202. The code for mutation analyses (python) and single-cell data analyses (R) is available here 10.5281/zenodo.12820202.

## References

[1] Haber, J. E. DNA repair: the search for homology. *BioEssays* **40**, 1700229 (2018). 10.1002/bies.201700229 PMC6238635 29603285

[2] Bétermier, M., Bertrand, P. & Lopez, B. S. Is non-homologous end-joining really an inherently error-prone process? *PLoS Genet.* **10**, e1004086 (2014). 24453986 10.1371/journal.pgen.1004086 PMC3894167

[3] McVey, M. & Lee, S. E. MMEJ repair of double-strand breaks (director’s cut): deleted sequences and alternative endings. *Trends Genet.* **24**, 529–538 (2008). 18809224 10.1016/j.tig.2008.08.007 PMC5303623

[4] Mladenov, E. & Iliakis, G. Induction and repair of DNA double strand breaks: the increasing spectrum of non-homologous end joining pathways. *Mutat. Res. Mol. Mech. Mutagen.* **711**, 61–72 (2011). 10.1016/j.mrfmmm.2011.02.005 21329706

[5] Sfeir, A. & Symington, L. S. Microhomology-mediated end joining: a back-up survival mechanism or dedicated pathway? *Trends Biochem. Sci.* **40**, 701–714 (2015). 26439531 10.1016/j.tibs.2015.08.006 PMC4638128

[6] Chan, S. H., Yu, A. M. & McVey, M. Dual roles for DNA polymerase Theta in Alternative End-joining repair of double-strand breaks in Drosophila. *PLOS Genet.* **6**, e1001005 (2010). 20617203 10.1371/journal.pgen.1001005 PMC2895639

[7] Grajcarek, J. et al. Genome-wide microhomologies enable precise template-free editing of biologically relevant deletion mutations. *Nat. Commun.* **10**, 4856 (2019). 31649251 10.1038/s41467-019-12829-8 PMC6813315

[8] He, M. D. et al. Efficient ligase 3-dependent microhomology-mediated end joining repair of DNA double-strand breaks in zebrafish embryos. *Mutat. Res. Mol. Mech. Mutagen.* **780**, 86–96 (2015). 10.1016/j.mrfmmm.2015.08.004 26318124

[9] Roerink, S. F., van Schendel, R. & Tijsterman, M. Polymerase theta-mediated end joining of replication-associated DNA breaks in C. Elegans. *Genome Res.* **24**, 954–962 (2014). 24614976 10.1101/gr.170431.113 PMC4032859

[10] Thyme, S. B. & Schier, A. F. Polq-mediated end joining is essential for surviving DNA double-strand breaks during early zebrafish development. *Cell. Rep.*10.1016/j.celrep.2016.03.072 (2016). 27149851 10.1016/j.celrep.2016.03.072 PMC5063659

[11] Truong, L. N. et al. Microhomology-mediated end joining and homologous recombination share the initial end resection step to repair DNA double-strand breaks in mammalian cells. *Proc. Natl. Acad. Sci.* **110**, 7720–7725 (2013). 10.1073/pnas.1213431110 PMC3651503 23610439

[12] van Schendel, R., Roerink, S. F., Portegijs, V., van den Heuvel, S. & Tijsterman, M. Polymerase Θ is a key driver of genome evolution and of CRISPR/Cas9-mediated mutagenesis. *Nat. Commun.* **6**, 7394 (2015). 26077599 10.1038/ncomms8394 PMC4490562

[13] Letsolo, B. T., Rowson, J. & Baird, D. M. Fusion of short telomeres in human cells is characterized by extensive deletion and microhomology, and can result in complex rearrangements. *Nucleic Acids Res.* **38**, 1841–1852 (2010). 20026586 10.1093/nar/gkp1183 PMC2847243

[14] Lin, T. T. et al. Telomere dysfunction and fusion during the progression of chronic lymphocytic leukemia: Evidence for a telomere crisis. *Blood* **116**, 1899–1907 (2010). 20538793 10.1182/blood-2010-02-272104

[15] Sinha, S., Villarreal, D., Shim, E. Y. & Lee, S. E. Risky business: Microhomology-mediated end joining. *Mutat. Res. Mol. Mech. Mutagen.* **788**, 17–24 (2016). 10.1016/j.mrfmmm.2015.12.005 PMC4887395 26790771

[16] Yan, C. T. et al. IgH class switching and translocations use a robust non-classical end-joining pathway. *Nature* **449**, 478–482 (2007). 17713479 10.1038/nature06020

[17] Mateos-Gomez, P. A. et al. Mammalian polymerase θ promotes alternative NHEJ and suppresses recombination. *Nature* **518**, 254–257 (2015). 25642960 10.1038/nature14157 PMC4718306

[18] Hogg, M., Sauer-Eriksson, A. E. & Johansson, E. Promiscuous DNA synthesis by human DNA polymerase θ. *Nucleic Acids Res.* **40**, 2611–2622 (2012). 22135286 10.1093/nar/gkr1102 PMC3315306

[19] Seki, M. et al. High-efficiency bypass of DNA damage by human DNA polymerase Q. *EMBO J.* **23**, 4484–4494 (2004). 15496986 10.1038/sj.emboj.7600424 PMC526458

[20] Mateos-Gomez, P. A. et al. The helicase domain of Polθ counteracts RPA to promote alt-NHEJ. *Nat. Struct. Mol. Biol.*10.1038/nsmb.3494 (2017). 29058711 10.1038/nsmb.3494 PMC6047744

[21] Wood, R. D. & Doublié, S. Genome Protection by DNA polymerase θ. *Annu. Rev. Genet.* **56**, 207–228 (2022). 36028228 10.1146/annurev-genet-072920-041046 PMC10351424

[22] Bermúdez-Guzmán, L. Pan-cancer analysis of non-oncogene addiction to DNA repair. *Sci. Rep.* **11**, 23264 (2021). 34853396 10.1038/s41598-021-02773-3 PMC8636604

[23] Higgins, G. S. et al. Overexpression of POLQ confers a poor prognosis in early breast cancer patients. *Oncotarget* **1**, 175–184 (2010). 20700469 10.18632/oncotarget.124 PMC2917771

[24] Lemée, F. et al. DNA polymerase θ up-regulation is associated with poor survival in breast cancer, perturbs DNA replication, and promotes genetic instability. *Proc. Natl. Acad. Sci.* **107**, 13390–13395 (2010). 20624954 10.1073/pnas.0910759107 PMC2922118

[25] Simsek, D. et al. DNA ligase III promotes alternative nonhomologous end-joining during chromosomal translocation formation. *PLOS Genet.* **7**, e1002080 (2011). 21655080 10.1371/journal.pgen.1002080 PMC3107202

[26] Gao, Y. et al. DNA ligase III is critical for mtDNA integrity but not Xrcc1-mediated nuclear DNA repair. *Nature* **471**, 240–244 (2011). 21390131 10.1038/nature09773 PMC3079429

[27] Lakshmipathy, U. & Campbell, C. The human DNA ligase III gene encodes Nuclear and mitochondrial proteins. *Mol. Cell. Biol.* **19**, 3869–3876 (1999). 10207110 10.1128/mcb.19.5.3869 PMC84244

[28] Perez-Jannotti, R. M., Klein, S. M. & Bogenhagen, D. F. Two forms of mitochondrial DNA ligase III are produced inXenopus Laevis oocytes *. *J. Biol. Chem.* **276**, 48978–48987 (2001). 11598119 10.1074/jbc.M107177200

[29] Puebla-Osorio, N., Lacey, D. B., Alt, F. W. & Zhu, C. Early embryonic lethality due to targeted inactivation of DNA ligase III. *Mol. Cell. Biol.* **26**, 3935–3941 (2006). 16648486 10.1128/MCB.26.10.3935-3941.2006 PMC1489003

[30] Westerfield, M. *The Zebrafish Book. A Guide for the Laboratory Use of Zebrafish (Danio Rerio)* (University of Oregon., 2007).

[31] Quan, F. B., Gaillard, A. L., Alejevski, F., Pézeron, G. & Tostivint, H. Urotensin II-related peptide (urp) is expressed in motoneurons in zebrafish, but is dispensable for locomotion in larva. *Peptides* **146**, 170675 (2021). 34655691 10.1016/j.peptides.2021.170675

[32] Hu, Y., Xie, S. & Yao, J. Identification of Novel reference genes suitable for qRT-PCR normalization with respect to the zebrafish developmental stage. *PLOS ONE*. **11**, e0149277 (2016). 26891128 10.1371/journal.pone.0149277 PMC4758726

[33] Bhat, P. et al. SLAMseq resolves the kinetics of maternal and zygotic gene expression during early zebrafish embryogenesis. *Cell. Rep.* **42**, 112070 (2023). 36757845 10.1016/j.celrep.2023.112070

[34] Sur, A. et al. Single-cell analysis of shared signatures and transcriptional diversity during zebrafish development. *Dev. Cell.*10.1016/j.devcel.2023.11.001 (2023). 37995681 10.1016/j.devcel.2023.11.001 PMC11181902

[35] Hao, Y. et al. Dictionary learning for integrative, multimodal and scalable single-cell analysis. *Nat. Biotechnol.* **42**, 293–304 (2024). 37231261 10.1038/s41587-023-01767-y PMC10928517

[36] Sposato, A. L. et al. Germ cell progression through zebrafish spermatogenesis declines with age. *Dev. Camb. Engl.* **151**, dev204319 (2024). 10.1242/dev.204319 PMC11607696 39470160

[37] Liu, Y. et al. Single-cell transcriptome reveals insights into the development and function of the zebrafish ovary. *eLife* 11, e76014 (2022). 10.7554/eLife.76014 PMC9191896 35588359

[38] Concordet, J. P. & Haeussler, M. C. R. I. S. P. O. R. Intuitive guide selection for CRISPR/Cas9 genome editing experiments and screens. *Nucleic Acids Res.* **46**, W242–W245 (2018). 29762716 10.1093/nar/gky354 PMC6030908

[39] Schneider, C. A., Rasband, W. S. & Eliceiri, K. W. NIH image to ImageJ: 25 years of image analysis. *Nat. Methods 2012*. **97**(9), 671–675 (2012). 10.1038/nmeth.2089 PMC5554542 22930834

[40] Tucker, B. & Lardelli, M. A Rapid apoptosis assay measuring relative Acridine Orange fluorescence in zebrafish embryos. *www Liebertpub com.* 10.1089=zeb.2007.0508 (2007). 10.1089/zeb.2007.0508 18041929

[41] Magoč, T. & Salzberg, S. L. FLASH: Fast length adjustment of short reads to improve genome assemblies. *Bioinformatics* **27**, 2957–2963 (2011). 21903629 10.1093/bioinformatics/btr507 PMC3198573

[42] R Core Team. *R: A Language and Environment for Statistical Computing* (R Foundation for Statistical Computing, 2021).

[43] Wickham, H. et al. Welcome to the tidyverse. *J. Open. Source Softw.* **4**, 1686 (2019).

[44] Wickham, H. *Ggplot2: Elegant Graphics for Data Analysis* (Springer-, 2016).

[45] Ninkovic, J. et al. Inhibition of neurogenesis at the zebrafish midbrain-hindbrain boundary by the combined and dose-dependent activity of a new hairy/E(spl)gene pair. *Development* **132**, 75–88 (2005). 15590746 10.1242/dev.01525

[46] Stenkamp, D. L. Chapter twenty-three - development of the Vertebrate Eye and Retina. in* Progress in Molecular Biology and Translational Science* (eds Hejtmancik, J. F. & Nickerson, J. M.) vol. 134 397–414 (Academic, (2015). 10.1016/bs.pmbts.2015.06.006 PMC5734922 26310167

[47] Wullimann, M. F. & Knipp, S. Proliferation pattern changes in the zebrafish brain from embryonic through early postembryonic stages. *Anat. Embryol. (Berl)*. **202**, 385–400 (2000). 11089930 10.1007/s004290000115

[48] Williams, A. B. & Schumacher, B. p53 in the DNA-Damage-repair process. *Cold Spring Harb Perspect. Med.* **6**, a026070 (2016). 27048304 10.1101/cshperspect.a026070 PMC4852800

[49] Lee, M. T., Bonneau, A. R. & Giraldez, A. J. Zygotic genome activation during the maternal-to-zygotic transition. *Annu. Rev. Cell. Dev. Biol.* **30**, 581–613 (2014). 25150012 10.1146/annurev-cellbio-100913-013027 PMC4303375

[50] Tucker, B. & Lardelli, M. A. Rapid apoptosis assay measuring relative acridine orange fluorescence in zebrafish embryos. *Zebrafish* **4**, 113–116 (2007). 18041929 10.1089/zeb.2007.0508

[51] Thyme, S. B. & Schier, A. F. Polq-mediated end joining is essential for surviving DNA double-strand breaks during early zebrafish development. *Cell. Rep.* **15**, 707–714 (2016). 27149851 10.1016/j.celrep.2016.03.072 PMC5063659

[52] van Overbeek, M. et al. DNA repair profiling reveals nonrandom outcomes at Cas9-Mediated breaks. *Mol. Cell.* **63**, 633–646 (2016). 27499295 10.1016/j.molcel.2016.06.037

[53] Kane, D. A. & Kimmel, C. B. The zebrafish midblastula transition. *Development* **119**, 447–456 (1993). 8287796 10.1242/dev.119.2.447

[54] McNally, J. R., Ames, A. M., Admiraal, S. J. & O’Brien, P. J. Human DNA ligases I and III have stand-alone end-joining capability, but differ in ligation efficiency and specificity. *Nucleic Acids Res.* **51**, 796–805 (2023). 36625284 10.1093/nar/gkac1263 PMC9881130

[55] Lu, G. et al. Ligase I and ligase III mediate the DNA double-strand break ligation in alternative end-joining. *Proc. Natl. Acad. Sci.* **113**, 1256–1260 (2016). 26787905 10.1073/pnas.1521597113 PMC4747774

[56] Maynard, S., Schurman, S. H., Harboe, C., de Souza-Pinto, N. C. & Bohr, V. A. Base excision repair of oxidative DNA damage and association with cancer and aging. *Carcinogenesis* **30**, 2–10 (2009). 18978338 10.1093/carcin/bgn250 PMC2639036

[57] Sallmyr, A., Bhandari, S. K., Naila, T., Tomkinson, A. E. & Mammalian DNA ligases; roles in maintaining genome integrity. *J. Mol. Biol.* **436**, 168276 (2024). 37714297 10.1016/j.jmb.2023.168276 PMC10843057

[58] Sallmyr, A., Rashid, I., Bhandari, S. K., Naila, T. & Tomkinson, A. E. Human DNA ligases in replication and repair. *DNA Repair.* **93**, 102908 (2020). 33087274 10.1016/j.dnarep.2020.102908 PMC8727047

[59] Gelot, C. et al. Polθ is phosphorylated by PLK1 to repair double-strand breaks in mitosis. *Nature* **621**, 415–422 (2023). 37674080 10.1038/s41586-023-06506-6 PMC10499603

[60] Shin, U. et al. Large-scale generation and phenotypic characterization of zebrafish CRISPR mutants of DNA repair genes. *DNA Repair.* **107**, 103173 (2021). 34390914 10.1016/j.dnarep.2021.103173

