# Supplementary material for: Dynamic interplay of cNHEJ and MMEJ pathways of DNA double-strand break repair during embryonic development in zebrafish
Source: Sci Rep. 2025 Feb 10;15:4886. doi: 10.1038/s41598-025-88564-6 (PMC11811205; doi:10.1038/s41598-025-88564-6)
Supplement: Supplementary file 1 — Supplementary Table S1 [file 41598_2025_88564_MOESM1_ESM.docx]

**Table S1**

| **Oligo Name** | **Sequence** |
| --- | --- |
|  |  |
| Genotyping |  |
| lig3_F1 | CAAGCGGATATGGCGGAGCA |
| lig3_R1 | GTCATTACGCTGCTGTGCCG |
| lig4_F1 | TGCAGCACTTCACAAGGACAAC |
| lig4_R1 | AATGTATGGTGTGAGGGAGCCC |
| lig4_F2 | AGCGACTACAGTTTCTTCCGTG |
| lig4_R2 | TGTTCACATGCTACTGAAACAAGTT |
| polq_F1 | GTGTCTGTGGCGAGAGAGAAGA |
| polq_R1 | GCGGGCAAGAAGATCCAAGTTT |
|  |  |
| qPCR |  |
| lig3_qpcrF1 | TGATGACAAGGACTGGAAGACG |
| lig3_qpcrR1 | GCTCTTGTCATTTTGGTTCGCT |
| lig4_qpcrF1 | GCGACTACAGTTTCTTCCGTGT |
| lig4_qpcrR1 | GAGGTGAATGAACGGAACTTGC |
| mre11a_qpcrF3 | CATGTGAACTCCTGTGTCAGTC |
| mre11a_qpcrR3 | TCTTGAACGTGTCCTCATCGTC |
| parp1_qpcrF1 | GGAGAGGTTGCATTAGGGAACA |
| parp1_qpcrR1 | TACTGTAGCTCTTGGGTCTGGA |
| polq_qpcrF2 | GCTGTTGCAAAGAAGTATGGCT |
| polq_qpcrR2 | CCAAGCCGATTACAGAAAACCG |
| prkdc_qpcrF1 | TGGAAGGACTGCCTCACAATAC |
| prkdc_qpcrR1 | CCCACAGAGTTATCCTTGCTGT |
| Rbbp8_qpcrF2 | CAGAACCATCATGCACCTTTCC |
| Rbbp8_qpcrR2 | AGCGTCTCCAAGTCTGTTCTG |
| xrcc4_qpcrF2 | ATGAGAAGGTGCAGAAGGACAT |
| xrcc4_qpcrR2 | GTAATGAGCTCCCGGATGACTT |
| xrcc5_qpcrF1 | GTGCTTGGCTTCACTAAACAGG |
| xrcc5_qpcrR1 | CGAATCAGAGAGGACAGAGCAA |
| xrcc6_qpcrF1 | AGTGATCTCGGCCTTCAGATTG |
| xrcc6_qpcrR1 | CCAGATCGACACCTTCTCCAAG |
| mob4_qpcrF1 | AAGAGTGCCCTGCCATTGATTA |
| mob4_qpcrR1 | AGTTTGGCCACAGATGATTCCT |
| lsm12b_qpcrF2 | GAGACTCCTCCTCCTCTAGCAT |
| lsm12b_qpcrR2 | GATTGCATAGGCTTGGGACAAC |
| Amplicons sequencing |  |
| uts2r5_1_F1 | CACCGAAGACTGAATTGAAGCTGT |
| uts2r5_1_R1 | TTACGCGCACGGAGGTGTTC |
| uts2r5_2_F1 | TCATGTGTTTGGTGGGCATAAC |
| uts2r5_2_R1 | TGACAGTCAGGATGAAGATGCTC |
| uts2r4_1_F1 | GAGTTGCAGGAGTAGTTTGGGT |
| uts2r4_1_R1 | CCCATCTCCCCGAAAAACCAAT |
| uts2r1_1_F1 | GCAAAACAAACTATAACTAATACAACCCA |
| uts2r1_1_R1 | CAACCAGCGTGTAGATATTTCCG |
| uts2r1_2_F1 | AGAGATGACCACTGTGTCTGTG |
| uts2r1_2_R1 | GCAAGTAGAGCAGATCAGCCA |
| uts2r2_1_F1 | ATTTGTCAAGTGTCCAAACACAGT |
| uts2r2_1_R1 | GATGGAGTGGCACATGACTACC |
| uts2r2_2_F1 | GAGTGTCGGGAAACGTCTACA |
| uts2r2_2_R1 | TGCACATCACCGTCAGAGTAAA |
| uts2r3_1_F1 | TTGCTCTCCAGGACAAATGGATA |
| uts2r3_1_R1 | GTATGCCGCCTTCTTGACATT |
| uts2r3_2_F1 | CCCCTTCTTTCTCTCCCTCTTTT |
| uts2r3_2_R1 | CGATACCGATCCAGACTCATAGC |
| *in situ* hybridization probes |  |
| lig3_ISH_F1 | ATGCGTGATGACAAGGACTGGA |
| lig3_ISH_R1 | CACTCCACACACCCAATCCTCA |
| polq_ISH_F1 | ATCTGGCTCTTCTAGGGGTGGA |
| polq_ISH_R1 | ATTTTGGTTTGGCTTCGGTGGG |
| parp1_ISH_F1 | TGATCCTGTGGGCCTCATTCTG |
| parp1_ISH_R1 | GCGTGAGTACAGCAGCTTAGAT |
| rbbp8_ISH_F1 | GGACACACACCTCGCAAAAGTT |
| rbbp8_ISH_R1 | TGTGTGAACAGCTCGACTCAGT |
| mre11a_ISH_F1 | TCTCGTCCCACTTCTGAGGTCT |
| mre11a_ISH_R1 | TGAACCTACTCAAATCGCTGCA |
| lig4_ISH_F1 | TGTGTCGCATTGGATCTGGCTA |
| lig4_ISH_R1 | CGCCTCAGTGTCTTTAGGTCCA |
| xrcc4_ISH_F1 | TCCGGCTTCGTCATCTTTCTGT |
| xrcc4_ISH_R1 | CTGGTCGCAGCTTTGGTTTCTT |
| xrcc5_ISH_F1 | CGTCAGTTCACTTTCCCCATGC |
| xrcc5_ISH_R1 | CCAACAAATAGAGGCTGCGCAA |
| xrcc6_ISH_F1 | TAGATCGCCTCAAACTGCACCA |
| xrcc6_ISH_R1 | TGTGACACCAGGCAAGACAGAA |
| prkdc_ISH_F1 | CAAGTTTCCTCGTCTGCTGCAG |
| prkdc_ISH_R1 | GGGCCTTGTGATTGTCTTCTGC |
|  |  |
| Guide RNAs |  |
| lig3_gRNA1 | GAGGAGTGAACAAGCGGATA |
| lig4_gRNA3 | TTCATGTTCTTAGGTTGGAA |
| lig4_gRNA4 | TTACGTTCACTACTATTAGA |
| polq_gRNA1 | GGAGGCAGGGATCAGGGTGG |
| polq_gRNA4 | TTGATGAGCTGCACATGGTT |
| 1#1 | TTTTCCACCATCGCCACTAT |
| 1#2 | GATGGTGCCAATGGTGAACG |
| 2#1 | CCACCGAACCAGAGTTGCTG |
| 2#2 | ACACACCACGAAAGGGATCG |
| 3#1 | GAGCAGTGGAAGCTACACTT |
| 3#2 | ACTGTCATACACAATGAAAG |
| 4#1 | GATGAAGACGTACATTGATC |
| 4#2 | CAACTGTTGGACTCGTAAAG |
| 5#1 | TAGCATGTCGTAAGAGGACG |
| 5#2 | AATACGTGCAGACCACGAAC |
