# Supplementary figures and images for: Dynamic interplay of cNHEJ and MMEJ pathways of DNA double-strand break repair during embryonic development in zebrafish
Source: Sci Rep. 2025 Feb 10;15:4886. doi: 10.1038/s41598-025-88564-6 (PMC11811205; doi:10.1038/s41598-025-88564-6)

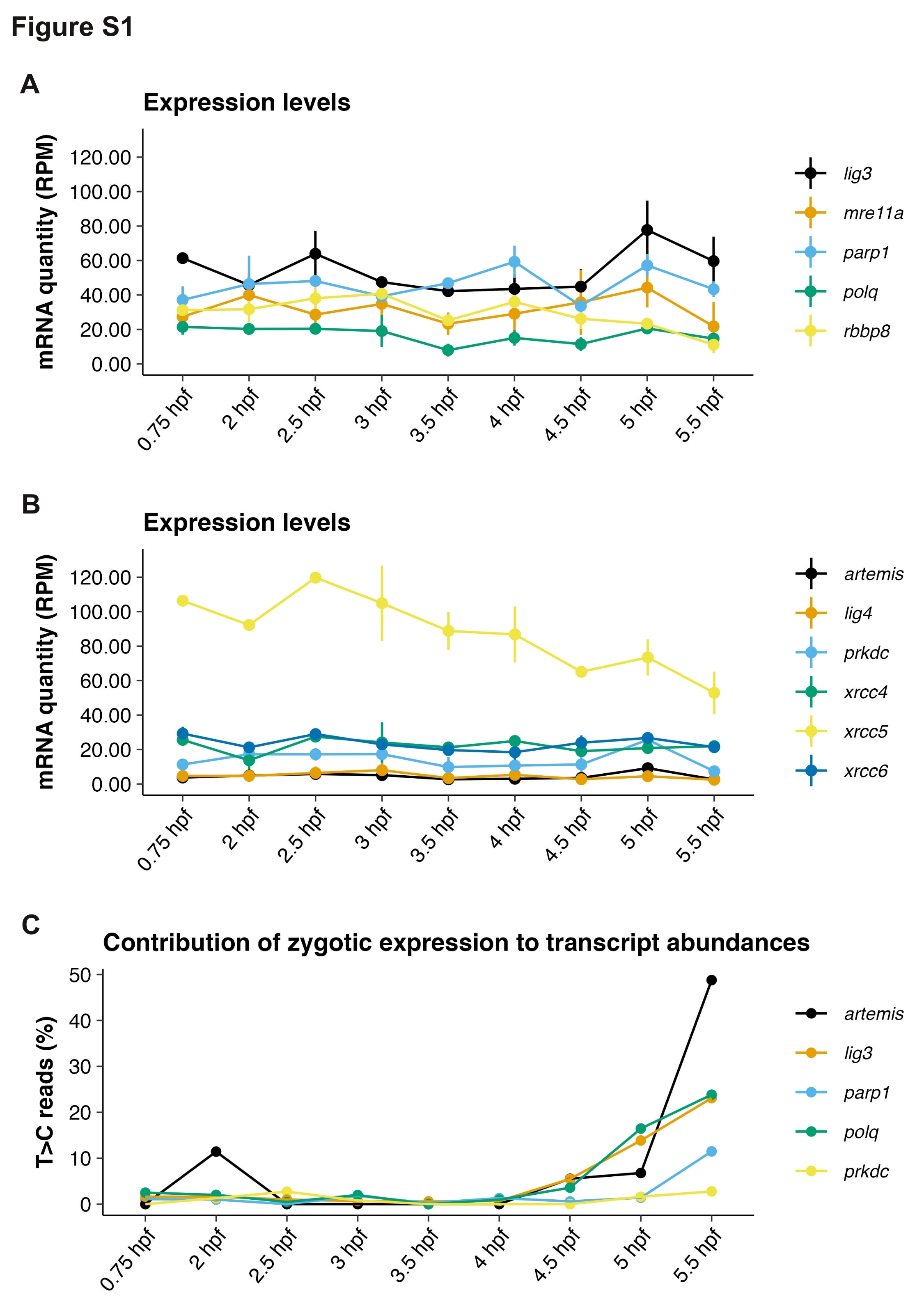

Supplement: Supplementary file 3 — Supplementary Figure S1 [file 41598_2025_88564_MOESM3_ESM.jpg]

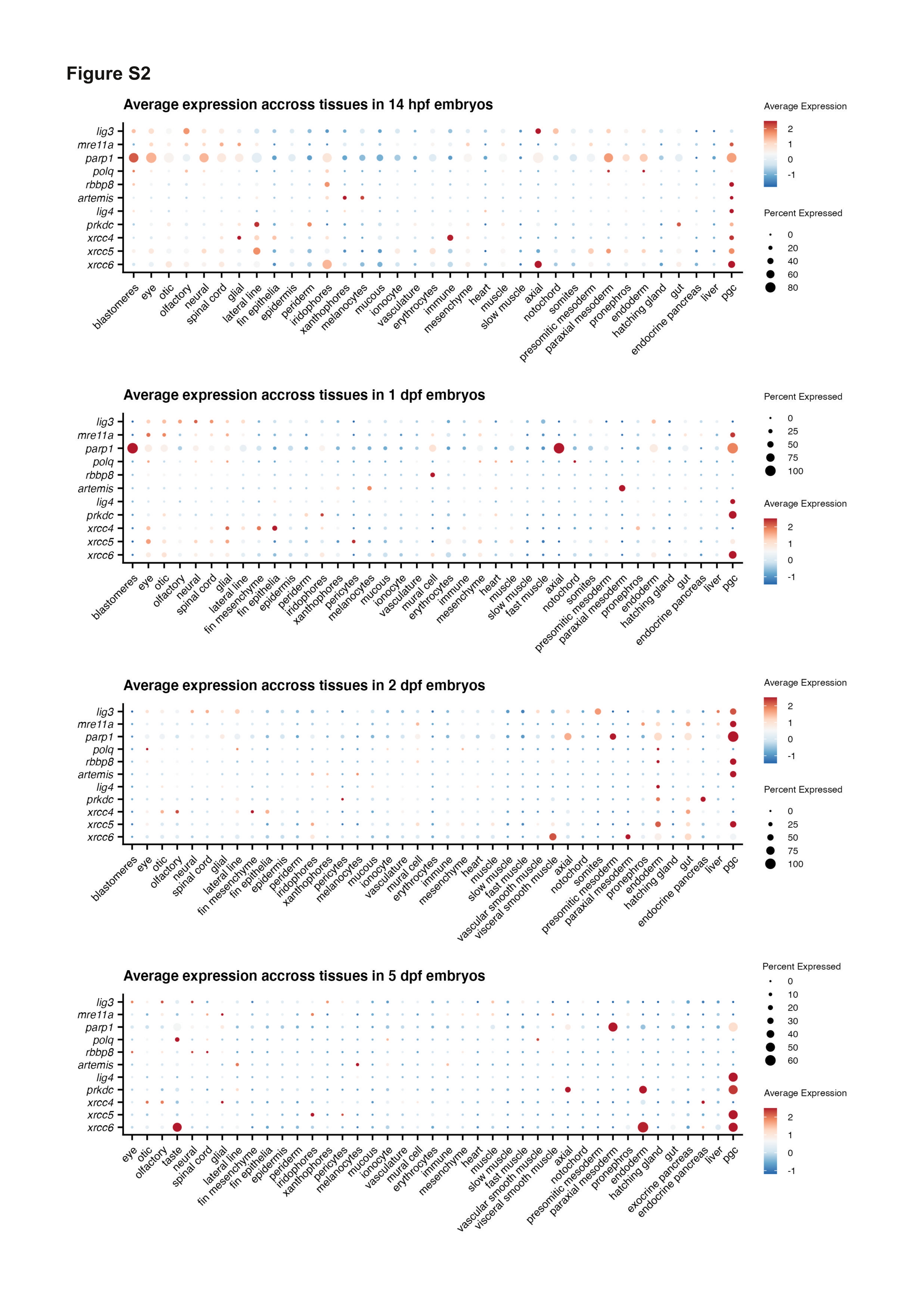

Supplement: Supplementary file 4 — Supplementary Figure S2 [file 41598_2025_88564_MOESM4_ESM.jpg]

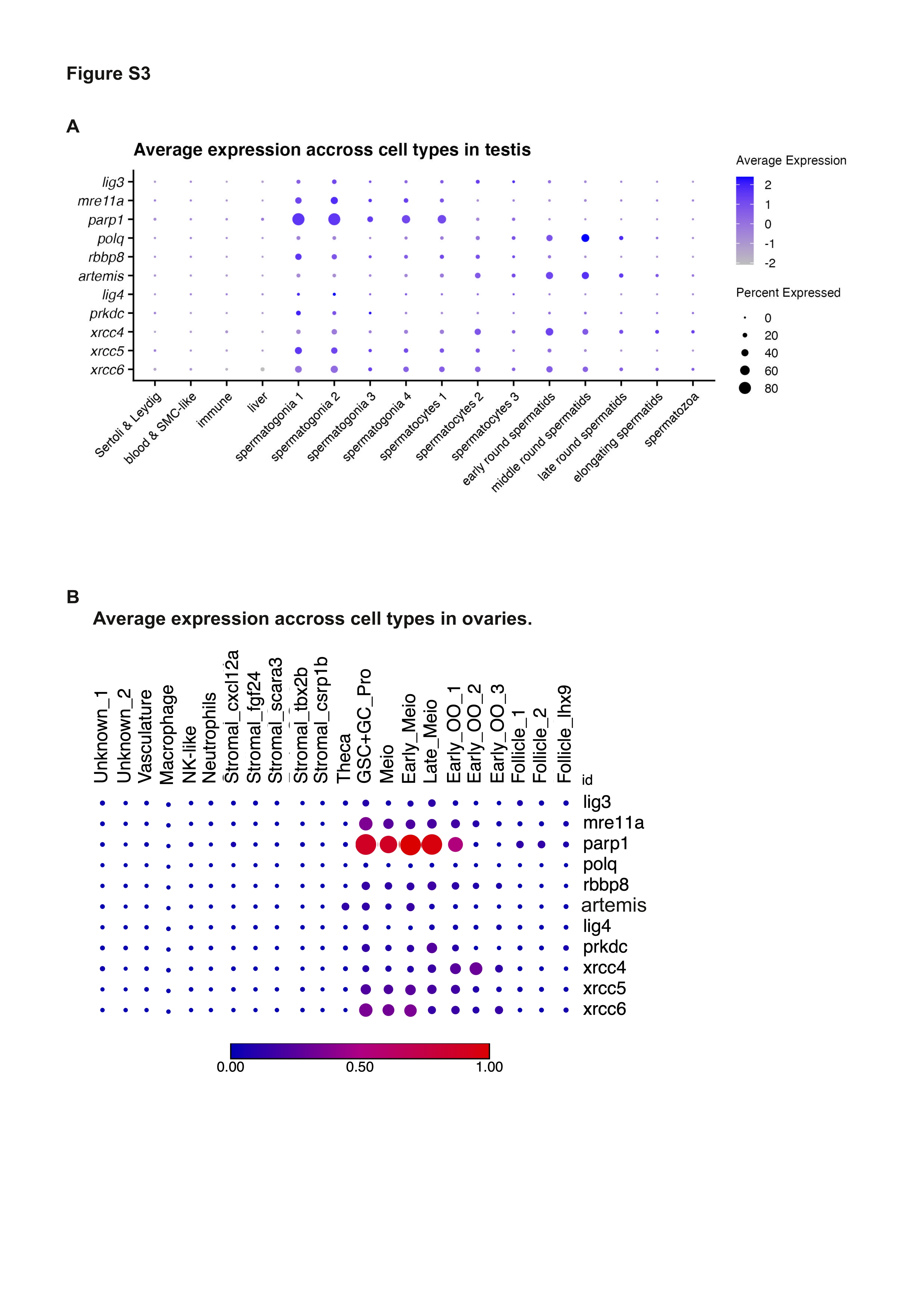

Supplement: Supplementary file 5 — Supplementary Material 2 [file 41598_2025_88564_MOESM5_ESM.jpg]
